# Advancing the fight against tuberculosis: integrating innovation and public health in diagnosis, treatment, vaccine development, and implementation science

**DOI:** 10.3389/fmed.2025.1596579

**Published:** 2025-08-25

**Authors:** Ayman Elbehiry, Eman Marzouk, Husam M. Edrees, Riyad AlShaqi, Abousree T. Ellethy, Feras Alzaben, Sulaiman Anagreyyah, Ahmad Algarni, Khalid Almuhaydili, Ibrahim Alotaibi, Abdulrahman Albaqami, Khalid Alamri, Mai Ibrahem, Abdulaziz M. Almuzaini, Falih Dhahri, Akram Abu-Okail

**Affiliations:** 1 Department of Public Health, College of Applied Medical Sciences, Qassim University, Buraydah, Saudi Arabia; 2 Department of Physiology, Faculty of Medicine, University of Tabuk, Tabuk, Saudi Arabia; 3 Defence Against Weapons of Destruction Department, Armed Forces Medical Services, Riyadh, Saudi Arabia; 4 Department of Basic Oral Sciences and Dental Education, College of Dentistry, Qassim University, Buraydah, Saudi Arabia; 5 Department of Food Service, King Fahad Armed Forces Hospital, Jeddah, Saudi Arabia; 6 Family Medicine Department, King Fahad Armed Hospital, Jeddah, Saudi Arabia; 7 Health Informatics Department, Prince Sultan Military Medical City, Riyadh, Saudi Arabia; 8 Education and Training Department, Prince Sultan Military College of Health Sciences, Dammam, Saudi Arabia; 9 Pharmaceutical Department, Prince Sultan Military Medical City, Riyadh, Saudi Arabia; 10 Department of Public Health, College of Applied Medical Science, King Khalid University, Abha, Saudi Arabia; 11 Department of Veterinary Preventive Medicine, College of Veterinary Medicine, Qassim University, Buraydah, Saudi Arabia; 12 Orthopedic Department, King Abdulaziz Hospital, Jeddah, Saudi Arabia; 13 Department of Pathology and Laboratory Diagnosis, College of Veterinary Medicine, Qassim University, Buraydah, Saudi Arabia

**Keywords:** tuberculosis, diagnostic innovation, therapeutic strategies, vaccine development, public health

## Abstract

Tuberculosis (TB) remains one of the leading causes of infectious disease mortality worldwide, increasingly complicated by the emergence of drug-resistant strains and limitations in existing diagnostic and therapeutic strategies. Despite decades of global efforts, the disease continues to impose a significant burden, particularly in low- and middle-income countries (LMICs) where health system weaknesses hinder progress. This comprehensive review explores recent advancements in TB diagnostics, antimicrobial resistance (AMR surveillance), treatment strategies, and vaccine development. It critically evaluates cutting-edge technologies including CRISPR-based diagnostics, whole-genome sequencing, and digital adherence tools, alongside therapeutic innovations such as shorter multidrug-resistant TB regimens and host-directed therapies. Special emphasis is placed on the translational gap—highlighting barriers to real-world implementation such as cost, infrastructure, and policy fragmentation. While innovations like the Xpert MTB/RIF Ultra, BPaLM regimen, and next-generation vaccines such as M72/AS01E represent pivotal progress, their deployment remains uneven. Implementation science, cost-effectiveness analyses, and health equity considerations are vital to scaling up these tools. Moreover, the expansion of the TB vaccine pipeline and integration of AI in diagnostics signal a transformative period in TB control. Eliminating TB demands more than biomedical breakthroughs—it requires a unified strategy that aligns innovation with access, equity, and sustainability. By bridging science with implementation, and integrating diagnostics, treatment, and prevention within robust health systems, the global community can accelerate the path toward ending TB.

## 1 Introduction

Tuberculosis (TB) is a bacterial disease primarily caused by *Mycobacterium tuberculosis* (*M. tuberculosis*) and related species (1). It remains a major global health concern due to its high morbidity and mortality rates (2). In 2022, TB was responsible for an estimated 1.3 million deaths, and projections suggest it could cause up to 31 million deaths in the coming years (3). Although TB primarily affects the lungs, it can also involve other organs, resulting in extrapulmonary manifestations (4, 5). Approximately 15–20% of TB cases present as extrapulmonary TB, especially among individuals co-infected with HIV (6). Furthermore, *M. tuberculosis* has been associated with tumor-like formations, with some studies linking pulmonary TB to lung neoplasms, emphasizing the importance of effective management to reduce this risk (7–12).

Transmission occurs through airborne particles expelled when an infected individual coughs or sneezes, making air a crucial vector for the spread of TB (13). Delayed treatment of active TB significantly increases the risk of transmission (14). Diagnosing TB can be particularly challenging due to misdiagnosis, non-specific symptoms, and limited laboratory capacity in many settings (15). The COVID-19 pandemic has exacerbated these challenges by overwhelming healthcare systems, interrupting TB services, and diverting essential resources. This disruption has led to delays in diagnosis and treatment, contributing to increased transmission and worsened clinical outcomes. Although the Global TB Report highlights partial recovery of TB services, the pandemic's residual impact continues to impede progress (16).

Additionally, the growing incidence of non-tuberculous mycobacterial infections, especially in elderly and immunocompromised individuals, complicates differential diagnosis and clinical management (17). Globally, ~23% of the population (95% CI: 20.4–26.4%), corresponding to 1.7–1.9 billion people, are estimated to harbor latent *M. tuberculosis* infection (LTBI), rather than active disease (18). In 2022, an estimated 7.5 million new TB cases and 1.5 million deaths were reported (16). Early detection remains vital for initiating timely treatment and curbing disease transmission (7, 19). According to the WHO Global Tuberculosis Report 2024, an estimated 10.8 million people (95% uncertainty interval [UI]: 10.1–11.7 million) developed active TB in 2023, corresponding to 134 new cases per 100,000 population (95% UI: 125–145) (20).

Since 2000, global efforts to combat TB have led to measurable progress. However, these gains were significantly undermined by the COVID-19 pandemic, which disrupted TB services worldwide. The reallocation of healthcare resources to COVID-19, combined with lockdowns and mobility restrictions, caused widespread delays in diagnosis and treatment. Furthermore, ongoing armed conflicts and deteriorating socioeconomic conditions—particularly in high-burden regions—have compounded these difficulties (21, 22). Economic instability, rising living costs, and declining public health funding have disproportionately affected impoverished communities already at heightened risk for TB (23).

According to WHO data, global TB case notifications dropped by 18% in 2020 due to limited access to health services. Although a partial rebound occurred in 2021, it failed to fully address the backlog of missed cases. Countries such as India, Indonesia, and the Philippines recorded the largest declines in notifications, resulting in a significant number of untreated cases and sustained community-level transmission. Between 2019 and 2021, TB-related deaths rose, with 1.6 million deaths reported in 2021 alone-−1.4 million among HIV-negative individuals and 187,000 among those living with HIV. These figures highlight the heightened vulnerability of HIV-positive individuals and the critical importance of uninterrupted TB services.

The pandemic also intensified pre-existing disparities in access to diagnostic and treatment tools. LMICs, which account for nearly 80% of TB cases globally, continue to face substantial challenges in adopting WHO-endorsed technologies (23). In 2022, TB incidence climbed to an estimated 10.6 million new cases—a 4.5% increase from 2020—sustaining the trend sparked by the pandemic. Simultaneously, drug-resistant TB remained a significant challenge, with ~410,000 new cases of rifampicin-resistant or multidrug-resistant TB reported (24). Studies indicate that MDR-TB disproportionately affects regions with low socio-demographic indices, adding complexity to control strategies (25).

Although molecular diagnostics such as GeneXpert MTB/RIF and line probe assays have transformed TB detection, their widespread implementation remains limited in many resource-constrained settings. Key obstacles include underdeveloped laboratory infrastructure, inefficient specimen transport systems, and shortages of trained personnel (26, 27). In contrast, high-income countries benefit from advanced diagnostic networks, enabling timely detection and robust drug resistance profiling that support effective patient care. Despite diagnostic advancements, only about 7.5 million of the estimated 10.6 million TB cases in 2022 were reported, reflecting a diagnostic gap of ~3.1 million cases. This persistent shortfall, worsened by pandemic-related disruptions, continues to delay treatment, perpetuate transmission, and increase mortality.

Effective TB control requires a multipronged approach that integrates conventional methods—such as microscopy and culture—with cutting-edge molecular diagnostics (28, 29). Clinical microbiology laboratories play a central role in diagnosis, and recent genetic technologies now allow for rapid identification of both the pathogen and its resistance profile. In countries where TB testing is predominantly conducted by general health or private sectors, it is essential to assess whether service expansion improves health outcomes or merely increases capacity. Evidence suggests that the quality of TB laboratory services, rather than their independence, is what determines their public health value (29).

Furthermore, distinguishing latent from active TB and ensuring equitable access to diagnostic services remain urgent priorities (30). Reliable drug susceptibility testing and a better understanding of the molecular mechanisms of resistance in *M. tuberculosis* are also critical. As multidrug-resistant and extensively drug-resistant TB pose growing threats, the need for improved detection and treatment strategies is more pressing than ever. Recent genetic innovations have accelerated the diagnosis of TB and its resistance traits. However, low-resource countries still urgently need affordable, rapid, and accurate diagnostic solutions (31). This review aims to provide a comprehensive overview of both standard and advanced diagnostic methods, while also exploring emerging therapeutic strategies to combat TB, with special emphasis on multidrug-resistant forms of the disease.

## 2 History and pathogenesis of TB

TB, caused by *M. tuberculosis*, remains one of the most lethal infectious diseases globally. First identified by Robert Koch in 1882 (32), the bacillus is transmitted via aerosols and primarily infects the lungs (pulmonary TB), although extrapulmonary forms also occur (33, 34). TB's ability to establish latent infection in an estimated two billion people worldwide contributes to its persistence (33). Latent TB infection can reactivate, particularly in immunocompromised individuals, including those with HIV, who have an 18-fold increased risk of disease progression (35, 36).

The infection begins when inhaled bacilli reach the alveoli and are phagocytosed by resident macrophages (37). Instead of being destroyed, *M. tuberculosis* manipulates the host immune system using the ESX-1 secretion system to release ESAT-6, facilitating escape from the phagosome into the cytoplasm (38, 39). The bacilli avoid degradation, inhibit phagosome maturation, and modulate cytokine responses, thereby creating an environment conducive to survival (40). This early evasion strategy is central to the pathogen's persistence and underlies the challenges in early TB diagnosis.

The host's immune response typically culminates in granuloma formation—a hallmark of TB pathogenesis—where bacilli are sequestered but not eradicated (41). Within these structures, *M. tuberculosis* can enter a dormant state, resisting immune clearance and pharmacological treatment (42). In individuals with compromised immunity, granulomas can break down, leading to bacterial reactivation, tissue necrosis, and contagious active TB (43, 44). Recent studies highlight that lipid metabolism, particularly the formation of foamy macrophages and caseous necrosis, plays a crucial role in long-term bacterial survival and disease progression (43, 45).

**Figure 1:**
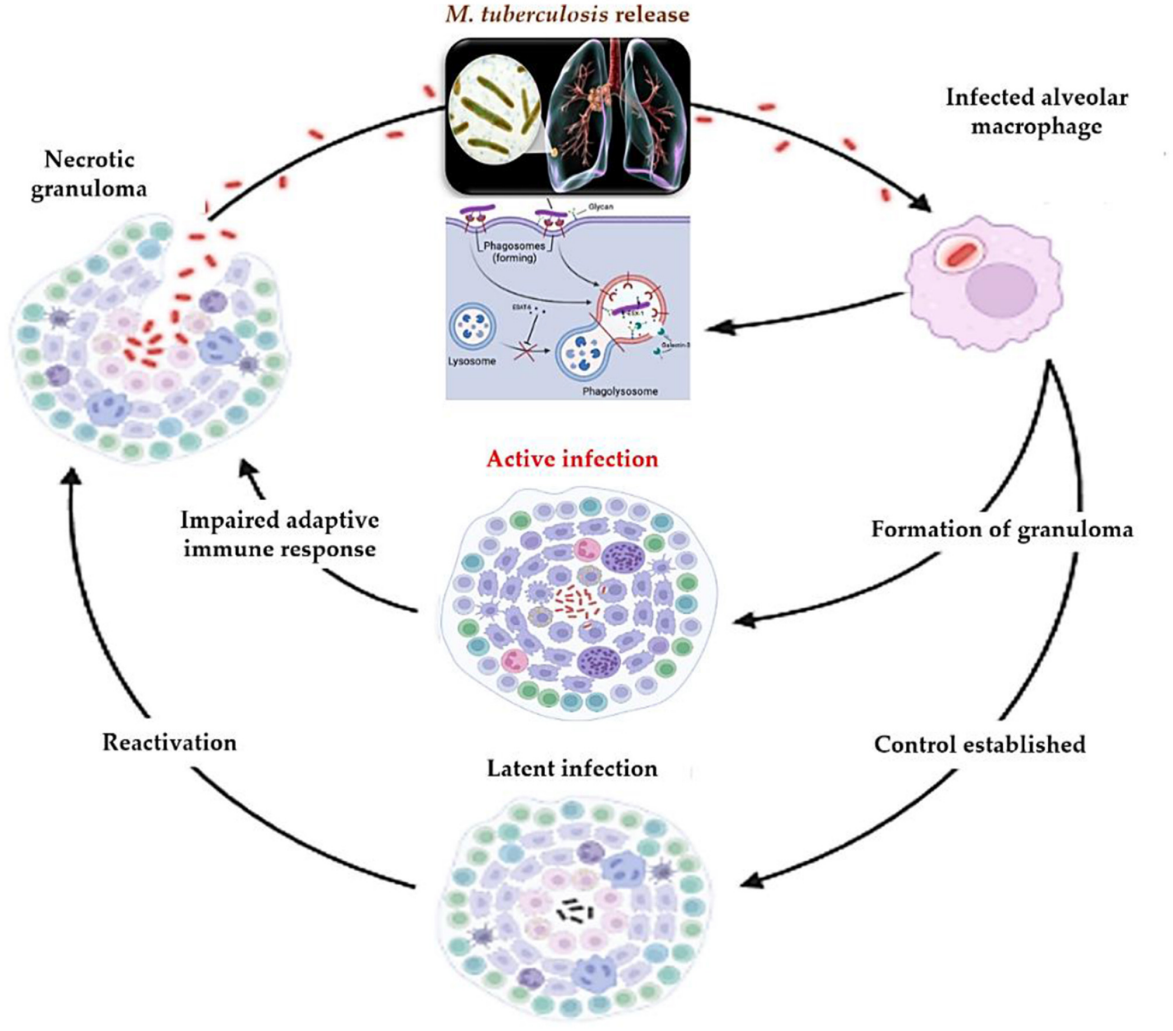
Pathogenesis of *M. tuberculosis*: from initial infection to reactivation. Following inhalation, *M. tuberculosis* is phagocytosed by alveolar macrophages, where it employs the ESX-1 secretion system to escape phagosomal destruction. This triggers granuloma formation as the host attempts to contain the infection. Control may be established, leading to latent infection, or the host may progress to active disease. Impaired immune responses or external stressors can disrupt granuloma integrity, leading to reactivation. Necrotic granulomas facilitate bacillary dissemination and renewed transmission.

Understanding TB pathogenesis is no longer solely of academic interest—it informs the development of new diagnostic tools and therapeutic strategies. For instance, insights into the ESX-1 system and granuloma biology have prompted the exploration of immunomodulatory therapies and biomarkers for early detection (46). Moreover, caseum-resident bacilli represent a significant challenge to drug penetration, guiding the design of new drug regimens with improved tissue distribution. In summary, TB pathogenesis illustrates a complex interplay between bacterial virulence and host immunity. A deeper mechanistic understanding is essential not only for accurate diagnosis—particularly distinguishing active from latent infection—but also for innovating treatment strategies that can overcome the barriers posed by immune evasion and granuloma-mediated bacterial persistence. The progression and immune interactions in TB pathogenesis are illustrated in Figure 1.

## 3 Innovations in tuberculosis diagnostics

**Figure 2:**
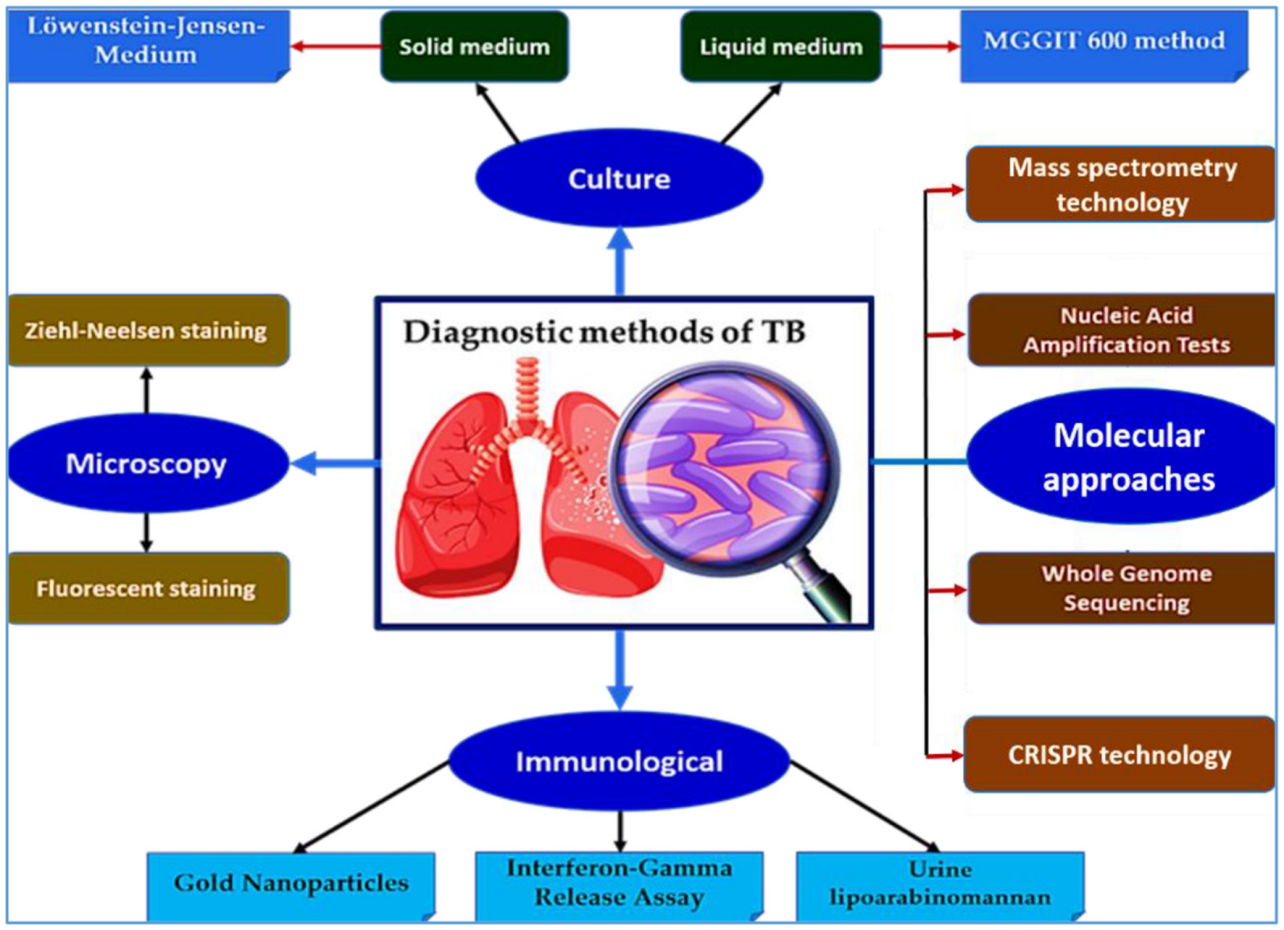
Overview of diagnostic methods for TB. This schematic illustrates the major diagnostic approaches for TB, including culture-based methods (solid and liquid media), microscopy (Ziehl-Neelsen and fluorescence staining), molecular diagnostics—such as nucleic acid amplification tests (NAATs), whole genome sequencing (WGS), CRISPR-based assays, and mass spectrometry—as well as immunological tools (e.g., interferon-gamma release assays, lipoarabinomannan (LAM) detection, and gold nanoparticles (GNPs)-based tests). These platforms enable early detection, resistance profiling, and improved disease management, especially in high-burden settings.

Effective TB control hinges on accurate, rapid diagnosis, yet the disease continues to be underdiagnosed, especially in resource-limited settings. While conventional methods such as smear microscopy and culture remain important, they lack sensitivity or are too slow for timely intervention. In recent years, molecular and next-generation diagnostic tools have dramatically enhanced the landscape of TB diagnostics, offering improved sensitivity, specificity, and speed. The main diagnostic platforms for TB, including conventional and modern approaches, are summarized in Figure 2.

### 3.1 Culturing-based mycobacterial detection

Culture remains the definitive standard for diagnosing *M. tuberculosis* infection and assessing drug susceptibility (47). Although *M. tuberculosis* exhibits a slow growth rate, dividing every 18–20 h, culture remains vital for confirming active disease and resistance patterns (48). Despite limitations in rapid detection (49), culture-based techniques are WHO-endorsed due to their high specificity and utility in confirming TB and its resistance profile (50). Culture-based methods outperform traditional microscopy, which detects only 10–100 bacteria per milliliter (51). Solid media such as Lowenstein-Jensen (LJ), Ogawa, and Middlebrook 7H10/7H11 are routinely employed for isolating *M. tuberculosis* (52). Middlebrook agars promote faster colony development, whereas LJ slopes allow reliable assessment of growth dynamics (53). However, the protracted growth period on solid media—typically up to 6 weeks—limits their practicality in urgent clinical scenarios.

To address this, automated liquid culture systems like the BACTEC MGIT 960 have been increasingly adopted. These systems improve time to detection—often reducing it to 10–15 days—and demonstrate enhanced sensitivity and specificity compared to solid culture (54–56). Liquid media such as 7H9 broth facilitate optimal bacterial proliferation and long-term strain preservation (57). Nonetheless, differentiating *M. tuberculosis* from non-tuberculous species, especially in HIV co-infected patients, requires supplemental biochemical or molecular confirmation (58). Although both media types differ in contamination rates and detection speed, the WHO recommends employing both in tandem where feasible (59). In resource-limited settings, this dual approach is often hindered by financial and logistical constraints (60). Despite these challenges, culture retains its importance in detecting persistent infections and evaluating recurrence risk (61). Recent innovations such as Thin-Layer Agar (TLA) culture offer promising alternatives. A 1-year study by Battaglioli et al. (62) in Indonesia demonstrated that TLA outperformed LJ media in both sensitivity and detection time, making it a potential tool for faster TB confirmation in high-burden areas.

The BACTEC MGIT 960 system supports rapid detection by culturing mycobacteria in a closed liquid environment with oxygen-sensitive fluorescent sensors (63, 64). A positive MGIT result, however, requires subsequent identification of the *M. tuberculosis* complex. Immunochromatographic tests detecting MPT-64 antigens are WHO-recommended tools that deliver rapid species-level identification with high sensitivity (98.1–98.6%) and specificity (99.2–100%) (65–67). While liquid culture offers reduced turnaround times (~2 weeks), it carries a higher risk of contamination (68). Drug susceptibility testing using MGIT 960 has shown high accuracy-−90% for isoniazid and 99.4% for rifampicin—with excellent specificity, outperforming nitrate reductase assays (69). Nonetheless, false negatives remain a concern in low-bacterial-load samples, especially during treatment monitoring. Cultures must therefore be conducted in well-equipped reference laboratories with trained personnel and biosafety precautions.

### 3.2 Microscopy

Microscopy remains a foundational tool in TB diagnosis, particularly in low-resource settings. For over a century, sputum smear microscopy has been the principal method for detecting *M. tuberculosis*, providing rapid, low-cost diagnostic support (29, 70). Despite being superseded in some regions by molecular assays, it remains essential for frontline diagnosis and infectiousness assessment in high-burden, low-income countries (71, 72). Two main staining approaches are widely used: the Ziehl-Neelsen (ZN) stain, which highlights acid-fast bacilli (AFB) in red against a blue background (73), and fluorescence microscopy (FM) using auramine dyes, which offers improved sensitivity but requires more specialized equipment (74). ZN microscopy is especially valuable in resource-limited environments where culture facilities are lacking (68, 75). It allows for the detection of high bacillary loads (>104 bacilli/mL), correlating with greater transmission risk (76). However, the technique suffers from low sensitivity, a high false-negative rate, and an inability to differentiate between viable/non-viable or tuberculous/non-tuberculous mycobacteria (29, 30).

A recent implementation of the ZEISS Axio Scan platform demonstrated a sensitivity of 97.06% and specificity of 86.44% for detecting and enumerating acid-fast bacilli, offering a valuable enhancement in diagnostic accuracy and operational efficiency for laboratory personnel (77). In pulmonary tissue specimens, the combination of fluorescent antibody labeling and laser confocal microscopy has proven especially effective in identifying *M. tuberculosis*, particularly when traditional ZN staining produces suboptimal results (78). Additionally, the integration of digital pathology systems, such as the Pat-Scan platform, with paraffin-embedded ZN-stained tissues, enables rapid and reliable identification and quantification of microorganisms, thereby reducing turnaround time for TB diagnosis (79).

While solid-state microscopy cannot reliably distinguish between viable and non-viable bacilli or differentiate *M. tuberculosis* from non-tuberculous mycobacteria, ZN staining continues to provide valuable morphological insight, especially in previously treated patients with AFB (80). The use of FM not only improves diagnostic sensitivity but also increases throughput and reduces labor demands (81). However, it is associated with limitations, including transient staining effects and the risk of false positives due to non-specific binding of fluorochrome dyes (82, 83).

To overcome these drawbacks, Light-Emitting Diode (LED) microscopy has emerged as a sustainable and practical alternative, particularly in resource-limited settings. Its extended battery life and low maintenance requirements make it suitable for peripheral laboratories where conventional light sources may be unreliable. In parallel, the fluorescein diacetate (FDA) staining method has gained attention for its ability to detect viable *M. tuberculosis* bacilli. This metabolic staining technique converts non-fluorescent FDA into green fluorescence within live cells, allowing for rapid assessment of bacterial viability and potentially predicting culture positivity within 1 h (84, 85). Fully automated imaging systems can now insert, focus, and scan smears, classifying them as positive or negative using algorithm-driven analysis (86).

Supporting these technical advances, Dzodanu et al. (87) compared ZN staining and FM in 100 suspected pulmonary TB patients at Kade Government Hospital. Of 200 sputum samples, 35.5% tested positive via FM, 23.2% via ZN staining, and 42% via the Xpert MTB/RIF assay. FM outperformed ZN in sensitivity (84.5% vs. 54.8%) with similar specificity (100%). Masali et al. (88) also reported superior performance for FM, detecting 42.3% positive cases vs. 18.2% by ZN, with FM achieving 98% sensitivity and a negative predictive value of 99%. These findings support FM as a more sensitive alternative to conventional staining techniques in TB diagnostics.

### 3.3 Molecular diagnostics for tuberculosis

Molecular diagnostics have significantly advanced TB detection by enabling rapid, sensitive, and specific identification of *M. tuberculosis* and associated drug resistance mutations. Unlike microscopy or culture—which are limited by either low sensitivity or long processing times—molecular tools offer results within hours, playing a vital role in early diagnosis and treatment decisions. This section outlines key molecular platforms used in TB diagnostics, including NAATs, real-time PCR (RT-PCR), WGS, mass spectrometry, and CRISPR-based diagnostics.

#### 3.3.1 Nucleic acid amplification tests (NAATs)

Over recent decades, various NAATs have been developed to improve the detection of *M. tuberculosis* complex (89–92). These tests show high specificity (74–99.3%) and variable sensitivity (64–100%), which can decrease to 40–84% in smear-negative samples or those with low bacillary loads (93). While many NAATs perform well in acid-fast smear-positive cases (~95% sensitivity), their sensitivity drops significantly in paucibacillary samples (94). Additionally, the presence of other microbes (e.g., non-tuberculous mycobacteria or fungal pathogens) may interfere with amplification, potentially leading to false-positive or false-negative outcomes (95, 96). Thus, enhancing diagnostic performance in smear-negative and extrapulmonary TB remains a key priority.

Insertion sequences (ISs) are widely used in NAATs to improve sensitivity (93). Targets such as IS986, IS987, IS1081, and particularly IS6110, are highly repetitive in the *M. tuberculosis* genome, allowing for effective amplification in multiplex PCR assays (97–99). However, certain strains (e.g., *M. bovis* BCG) may harbor few or no IS6110 copies, which may reduce test sensitivity. The Xpert MTB/RIF Ultra assay, endorsed by WHO, incorporates IS6110 and IS1081 as targets and is now widely used in clinical practice. This cartridge-based test detects TB DNA and rifampicin resistance with a sensitivity of 87.5% and a detection limit of 15.6 CFU/mL (100, 101). In lung specimens, it achieves 88% sensitivity and 96% specificity; in extrapulmonary TB, it yields 98.5% sensitivity and 97% specificity (100, 102). However, its sensitivity decreases to 78.9% in smear-negative samples. Despite its clinical utility, its cost (~$9.98/cartridge) poses challenges in low-resource settings (103), prompting a shift toward in-house real-time PCR protocols that are more affordable and adaptable.

**Figure 3:**
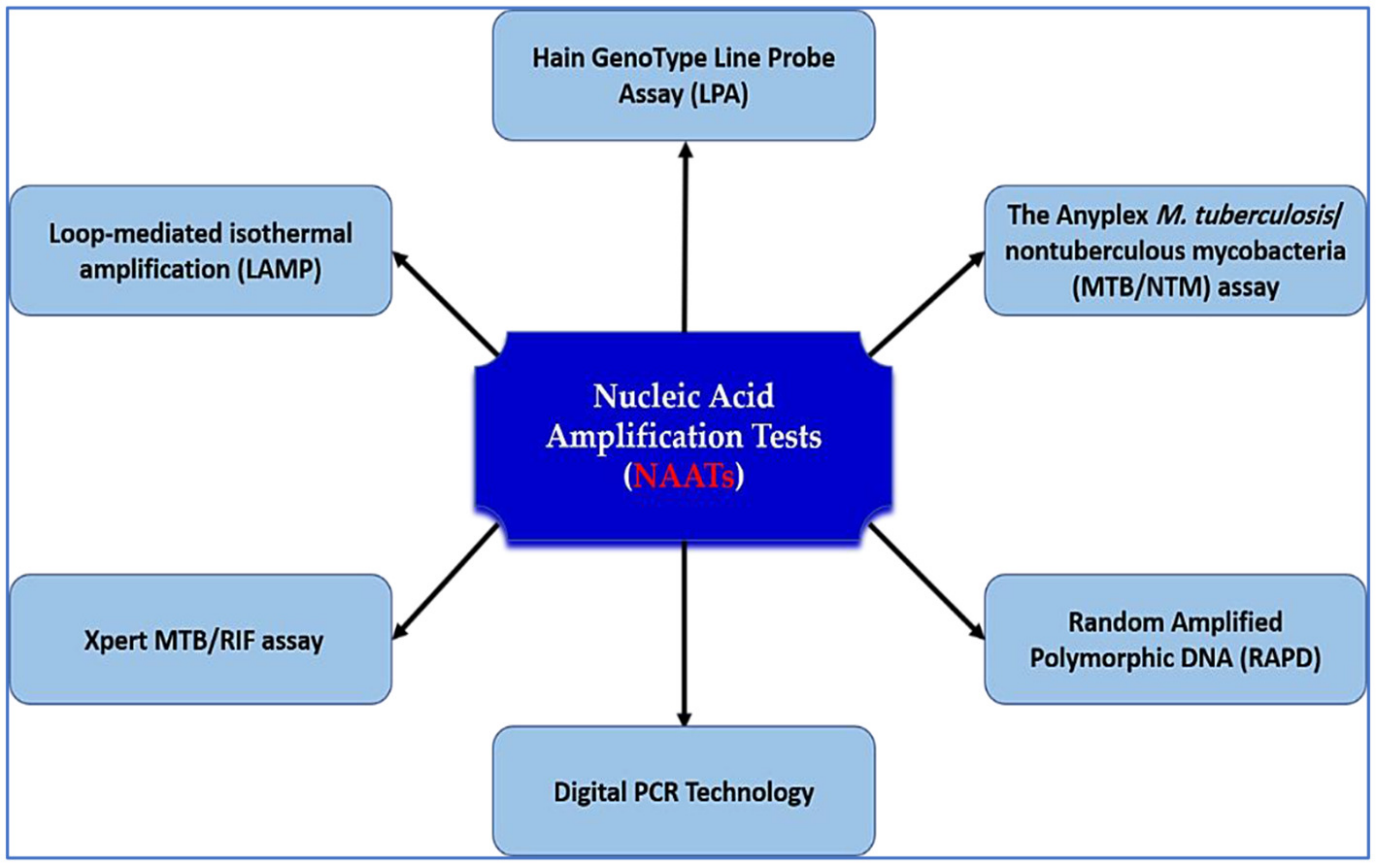
NAATs for tuberculosis diagnosis. This schematic illustrates the major nucleic acid amplification platforms used for TB detection, including Loop-mediated isothermal amplification (LAMP), Xpert MTB/RIF, digital PCR, Hain GenoType Line Probe Assay, Anyplex MTB/NTM assay, and RAPD. These assays improve diagnostic accuracy, speed, and resistance profiling, and are central to early TB case detection and treatment guidance.

Nested PCR, a subtype of NAAT, improves analytical sensitivity by using two rounds of amplification. Although effective for extrapulmonary TB (sensitivity: 72.2% for blood/urine vs. 33.3% for pleural fluid) (104–106), it is labor-intensive, prone to contamination, and costly. To overcome these drawbacks, single-tube nested PCR was developed, incorporating outer primers with higher annealing temperatures than inner primers. This method offers a simplified workflow and enhanced accuracy—achieving up to 89% sensitivity in pulmonary TB and 42% in extrapulmonary forms. Notably, single-tube nested RT-PCR has demonstrated even higher performance, with 97.2% sensitivity and 99.7% specificity (107). In a comparative analysis, Choi et al. reported 94.6% sensitivity for IS6110 RT-PCR and 100% for single-tube nested RT-PCR in sputum samples (108). Despite these advances, diagnostic performance for smear-negative and extrapulmonary TB remains suboptimal, reinforcing the urgent need for more robust, accessible, and affordable molecular platforms. Figure 3 illustrates the standard NAATs currently employed in TB diagnostics and their role in expediting accurate clinical decision-making.

##### 3.3.1.1 Loop-mediated isothermal amplification (LAMP)

LAMP, developed by Eiken Chemical Co., Ltd. (Tokyo, Japan), is an innovative nucleic acid amplification technique for TB detection that has been endorsed by the WHO (109). LAMP offers a cost-effective alternative to the Xpert MTB/RIF assay, with studies in China reporting diagnostic cost savings of 50–70% (110, 111). One of its major advantages is the visual readout of results, eliminating the need for sophisticated equipment and enabling deployment in decentralized, resource-limited settings. Multiple studies in China have evaluated LAMP's diagnostic performance for pulmonary TB, though reported accuracy has varied (112–114). Lin et al. (109) conducted a multicenter evaluation using respiratory samples from suspected TB patients across six sites in China between June 2018 and December 2019. LAMP showed good concordance with Xpert MTB/RIF, fluorescent smear microscopy, and BACTEC MGIT 960, with a sensitivity of 78.6% and specificity of 88.7%. The study concluded that LAMP is a rapid, user-friendly, and affordable alternative for TB detection in clinical practice.

Further validation came from Zaber et al. (115), who assessed a multiplex LAMP assay in 130 sputum samples in Bangladesh. Compared to qPCR, which detected *M. tuberculosis* in 56.92% of cases, LAMP identified 53.85%. LAMP demonstrated a sensitivity of 95% and specificity of 81.4% vs. culture, outperforming ZN staining by 16.93% and FM by 13.08%. Importantly, LAMP failed to detect non-tuberculous mycobacteria that were identified by qPCR in 7.69% of cases, suggesting high specificity for *M. tuberculosis*. These findings support LAMP as a WHO-recommended, low-cost, and effective diagnostic tool for TB control, especially in LMICs aiming to meet End TB Strategy targets by 2035 (115).

##### 3.3.1.2 Xpert MTB/RIF assay

The emergence of multidrug-resistant *(*MDR*) M. tuberculosis* strains has underscored the urgency for rapid diagnostic tools. In response, the Centers for Disease Control and Prevention (CDC) recommends integrating molecular diagnostics with traditional testing to overcome the limitations of conventional culture-based methods. The Xpert MTB/RIF assay (Cepheid, Sunnyvale, CA, USA) represents a transformative advancement, enabling direct detection of *M. tuberculosis* and rifampicin resistance from clinical specimens in under 2 h (116). In Brazil, the National Tuberculosis Control Program introduced the Xpert platform in 92 high-burden municipalities between 2014 and 2015. Subsequently, the Clinics Hospital of the University of São Paulo adopted the Xpert MTB/RIF assay for diagnosing both pulmonary and extrapulmonary TB.

In a study by Feliciano et al. (117), 2,148 respiratory samples collected between 2015 and 2018 were analyzed. The assay demonstrated a sensitivity of 94%, specificity of 98%, positive predictive value (PPV) of 89%, and negative predictive value (NPV) of 99%. Agreement with phenotypic drug susceptibility testing was 94.1%, while concordance with WGS was 78.9%. More recently, Terzi et al. (118) evaluated the performance of Xpert MTB/RIF in 2,082 specimens (1,526 respiratory and 556 non-respiratory). *M. tuberculosis* was cultured in 153 samples (7.3%), while 203 (9.7%) were Xpert-positive. The assay achieved 89.5% sensitivity, 96.6% specificity, 67.5% PPV, and 99.1% NPV. Its speed, simplicity, and ability to detect resistance in a single step make it a vital tool in TB diagnostics, especially in high-burden and resource-constrained settings.

##### 3.3.1.3 Line probe assay (LPA)

Line Probe Assays (LPAs), endorsed by the WHO in 2008, enable rapid molecular detection of *M. tuberculosis* and assessment of resistance to rifampicin and isoniazid—the two cornerstone drugs in TB treatment (119). A widely used commercial version was originally developed by Hain Lifescience (now acquired by Bruker). Based on DNA-STRIP technology, LPA improves diagnostic speed and precision compared to conventional phenotypic methods, though it requires greater technical expertise than the Xpert MTB/RIF assay (120, 121). The second-generation LPA (version 2) accommodates a wider variety of sample types and provides detailed resistance profiles, including detection of both low- and high-level resistance mutations (122).

In a 2024 study, Nandwani et al. (123) tested 196 sputum samples from suspected pulmonary TB patients using LPA. TB was confirmed in 104 positive cases. Sensitivity for smear-negative samples varied by target: 47.36% for TB detection, 72.72% for rifampicin resistance, and 88.88% for isoniazid resistance, with specificities ranging from 86.96% to 95.65%. In smear-positive cases, LPA achieved high sensitivity-−89.09% for TB, 95.83% for rifampicin resistance, and 98.07% for isoniazid resistance—with specificities exceeding 98% for all targets. These results support the use of LPA as a reliable and efficient tool for first- and second-line drug resistance detection, particularly in smear-positive patients, and as a complement to other rapid molecular tests in TB diagnostic algorithms.

##### 3.3.1.4 Anyplex MTB/NTM real-time detection assay

The Anyplex™ MTB/NTM assay (Seegene Inc., South Korea) utilizes real-time PCR with dual priming oligonucleotide (DPO™) technology to simultaneously detect *M. tuberculosis* complex and differentiate it from nontuberculous mycobacteria, offering enhanced specificity and multiplexing capabilities. It targets highly specific genetic markers, including the insertion sequence IS6110 (99) and the MPB64 gene (124), allowing for precise identification of clinically relevant mycobacterial species. In a large-scale evaluation by Sawatpanich et al. (125), the assay was applied to 9,575 clinical specimens. The test demonstrated a sensitivity of 79.7% and specificity of 94.5% for detecting MTBC, while for NTM, sensitivity was lower at 44.9% but specificity remained high at 97.7%. Among AFB smear-positive samples, the assay achieved significantly improved sensitivity-−97.7% for MTBC and 80% for NTM. These results support the utility of Anyplex MTB/NTM as a rapid and accurate tool, particularly valuable in high-throughput laboratory settings where distinguishing MTBC from NTM is crucial for patient management.

In parallel, Random Amplified Polymorphic DNA (RAPD)—also known as Arbitrary Primed PCR (AP-PCR)—is a molecular fingerprinting method that requires no prior sequence information. By employing randomly selected primers ranging from 5 to 50 base pairs, RAPD generates DNA profiles that can reveal inter-strain polymorphisms (126). Although some variability in results is attributed to technical reproducibility concerns (127), RAPD remains a valuable tool for analyzing strain diversity among NTM species. For instance, RAPD has been successfully used to genotype *M. abscessus* and *M. chelonae*, two species prone to DNA fragmentation during electrophoresis and difficult to analyze via pulsed-field gel electrophoresis (PFGE) (128). Furthermore, RAPD has proven effective in characterizing genetic diversity within other clinically significant NTMs such as *M. phocaicum, M. gordonae, M. szulgai*, and *M. malmoense* (129–132). The method's affordability and simplicity make it particularly appealing for resource-constrained laboratories aiming to monitor mycobacterial diversity and trace epidemiological patterns.

##### 3.3.1.5 Droplet digital polymerase chain reaction (ddPCR)

Droplet digital polymerase chain reaction (ddPCR) is an advanced molecular technique that enables absolute quantification of nucleic acids without requiring a standard curve (133). This method partitions the sample into thousands of nanoliter-sized droplets, where amplification occurs independently, improving detection sensitivity and reducing variability. ddPCR has emerged as a powerful tool for detecting low-abundance genetic targets, making it particularly suitable for infectious diseases such as tuberculosis (134). Recent studies have demonstrated the utility of ddPCR in TB diagnostics. Devonshire et al. (135) assessed ddPCR performance using *M. tuberculosis* DNA templates and confirmed its robustness and accuracy in quantifying bacterial DNA. In follow-up research, ddPCR successfully identified *M. tuberculosis* in artificially prepared sputum specimens, supporting its applicability in clinical settings (136). The method showed strong reproducibility and precision, even in samples with low bacterial loads.

Due to its superior sensitivity, ddPCR holds promise for various TB-related applications, including early diagnosis, quantification of drug resistance mutations, and monitoring of bacterial burden during treatment (137). Future innovations are expected to yield minimally invasive, rapid, and highly accurate ddPCR-based platforms for the detection of *M. tuberculosis*-specific genetic sequences, which could substantially improve disease surveillance and management strategies.

#### 3.3.2 Whole genome sequencing (WGS)

WGS has emerged as a transformative tool for tuberculosis diagnostics, offering unparalleled resolution in identifying *M. tuberculosis* complex strains and their resistance profiles. Thanks to reduced costs and rapid technological advances, WGS is now transitioning from research laboratories into clinical workflows for TB detection, drug resistance prediction, and epidemiological surveillance (138). WGS surpasses traditional genotyping techniques such as PCR and microarrays by enabling comprehensive analysis of nearly all genomic mutations associated with drug resistance, as well as differentiating closely related *Mycobacterium* subspecies (139). It offers significant value in tracking transmission chains and understanding pathogen evolution (140). Advances in sequencing platforms, including Illumina MiSeq™ and HiSeq 4000, have greatly increased throughput—generating between 15 and 1,500 gigabytes of sequence data—making WGS increasingly cost-effective in diagnostic settings (141).

The utility of WGS has been underscored by several key studies (142–144). The WHO issued a practical guide in 2018 promoting WGS to characterize treatment-resistant TB strains, highlighting platforms such as Ion Personal Genome Machine^®^, Nanopore MinION^®^, and GeneReader. In a large-scale study, Campbell et al. (145) sequenced nine drug-resistance loci in 314 clinical isolates, reporting a sensitivity of 90.8% and specificity of 94.7% for multidrug resistance, but only 40% sensitivity for extensively drug-resistant (XDR) strains. These findings highlight the promise and current limitations of WGS, especially for detecting rare resistance mutations (63). Emerging research illustrates that WGS can uncover low-level resistance mutations often missed by conventional phenotypic drug susceptibility tests (DST). For example, genome analysis has identified ethambutol resistance-associated mutations that remain undetected in phenotypic assays (146). Similarly, certain isoniazid-resistant isolates may yield negative WGS results, reflecting challenges in correlating genotype and phenotype (147).

In a study by Sun et al. (148), WGS was evaluated in newly diagnosed multidrug-resistant TB cases in China. The method showed high concordance with phenotypic DST for amikacin/kanamycin and rifampicin (97.7%) but lower agreement for rifabutin and ethambutol (67.2% and 79.1%, respectively). Pyrazinamide resistance-associated mutations were detected in 27.9% of isolates. No resistance mutations were found for newer drugs such as linezolid, bedaquiline, or clofazimine, affirming WGS's role in guiding individualized therapy in high-burden settings.

Recognizing the diagnostic potential of next-generation sequencing, the WHO issued guidelines in October 2023 to support its integration into national TB programs, particularly for tracking drug-resistant strains. However, real-world implementation faces numerous barriers. Vogel et al. (149) reported logistical and financial challenges in Kyrgyzstan, while Ness et al. (150) and others have emphasized the need for robust infrastructure, expert training, and standardized quality control measures (151, 152). Recent innovations, such as direct sputum WGS (bypassing culture), show promise for reducing turnaround times (153, 154). Yet, obstacles such as low DNA yield, high costs, limited database access, and technical complexity continue to hinder widespread clinical adoption (155). Despite these constraints, WGS remains a powerful tool in the global fight against TB, with future applications likely to enhance precision diagnostics and public health interventions.

### 3.4 Immunological approaches

#### 3.4.1 Gold nanoparticles (GNPs)

GNPs have emerged as promising tools in TB diagnostics due to their nanoscale size, ease of synthesis, high stability, and biocompatibility (156, 157). Their unique optical behavior—particularly localized surface plasmon resonance—is influenced by particle size, shape, and interparticle spacing, allowing colorimetric detection without the need for complex instrumentation (158). In point-of-care (POC) platforms, GNPs function as optical tags for antigen–antibody detection, offering a rapid, visual signal for TB diagnosis (159, 160). GNPs possess the unique ability to change color via a mechanism known as localized surface plasmon resonance, which is influenced by the particle's shape, size, and local refractive index (161, 162). To date, only one GNP-based diagnostic platform—the TB-LAMP assay developed by Eiken Chemical Co.—has received endorsement by the WHO. This approval was issued in 2016, recommending its use as a molecular alternative to sputum smear microscopy in resource-limited settings for the detection of *M. tuberculosis* (163).

Several studies have demonstrated the diagnostic efficacy of GNPs in TB. Becerra et al. (164) developed a plasmonic system using GNPs coated with mycobacterial lipid glycans to detect anti-lipid antibodies in patient sera. The system showed measurable shifts in LSPR (up to 2 nm), enabling sensitive antigen–antibody detection confirmed across multiple clinical samples (165). Other biosensor platforms targeting the *M. tuberculosis* IS6110 gene reported sensitivities ranging from 84.7% to 100% and specificities approaching 100%, with detection limits from 5 pg to 81 ng per 25 μL reaction (166–170).

Dahiya et al. (171) developed a magnetic bead-coupled GNP immuno-PCR (MB-GNP-I-PCR) assay for detecting TB antigens in clinical fluids, demonstrating 89.3% sensitivity for pulmonary TB and 78.1% for extrapulmonary TB, with specificity exceeding 97.9%. This approach outperformed conventional assays such as Magneto-ELISA and GeneXpert. Similarly, Kooti et al. (158) validated a GNP biosensor assay that reliably detected *M. tuberculosis* in sputum samples, underscoring its utility as a simple and scalable method adaptable to various diagnostic settings. In summary, GNP-based diagnostics represent a powerful and cost-effective tool for rapid TB detection. While further standardization is needed, their sensitivity, adaptability, and compatibility with low-resource environments support their future integration into global TB control programs.

#### 3.4.2 Interferon gamma release assay (IGRA)

Interferon-gamma release assays (IGRAs) have emerged as valuable tools in the diagnosis of LTBI, offering improved specificity over the traditional tuberculin skin test (TST). The TST is prone to false-positive results, particularly in individuals vaccinated with Bacille Calmette-Guérin (BCG) or exposed to non-tuberculous mycobacteria (172). IGRAs circumvent this issue by utilizing M. tuberculosis-specific antigens, such as ESAT-6 and CFP-10, which are absent from BCG strains and most environmental mycobacteria. Two widely used IGRAs are the QuantiFERON-TB Gold Plus (QFT-Plus) and T-SPOT.TB, developed by Qiagen and Oxford Immunotec, respectively (173, 174). QFT-Plus, a fourth-generation IGRA, detects IFN-γ released by T cells in response to *M. tuberculosis* complex-specific antigens, including *M. tuberculosis, M. bovis*, and *M. africanum* (175). It serves as a more specific alternative to the traditional TST for identifying latent TB infection. Compared to earlier versions like QuantiFERON-TB Gold In-Tube (QFT-GIT), QFT-Plus incorporates both CD4+ and CD8+ T-cell responses, potentially enhancing sensitivity.

Although IGRA results can indicate *M. tuberculosis* infection, they do not distinguish between latent and active TB (176). Furthermore, variables such as overnight incubation and host immune status may affect test reliability, especially in immunocompromised individuals (177, 178). In a 2019 study by Hong et al. comparing 33 active TB patients and 57 controls with LTBI, QFT-Plus demonstrated a sensitivity of 93.9% and a specificity of 92.6%, while QFT-GIT showed identical sensitivity but slightly higher specificity at 100%. Notably, IFN-γ levels were lower in latent TB cases using QFT-Plus, although the distinction between active and latent TB remained inconclusive (179). Similarly, Venkatappa et al. (180) evaluated the concordance between QFT-Plus, QFT-GIT, T-SPOT.TB, and TST across 506 high-risk individuals in a multicenter study. QFT-Plus and QFT-GIT exhibited 94% overall agreement, with 19% positivity and 75% negativity, reinforcing their equivalence in clinical utility. Despite their diagnostic strengths, IGRAs face limitations in differentiating disease stages and in implementation across low-resource settings. Nonetheless, they remain a vital component of TB screening protocols, especially in BCG-vaccinated populations and high-risk groups.

#### 3.4.3 Urine lipoarabinomannan assay (LAM)

Urine-based antigen detection offers a noninvasive diagnostic option for TB, eliminating the need for aerosol-generating procedures during specimen collection (181). One promising biomarker in this approach is LAM, a glycolipid component of the *M. tuberculosis* cell wall that can be excreted in urine. Although the exact mechanism of LAM excretion remains unclear, it is hypothesized to result from bacterial degradation during infection (182–184). The Determine™ TB LAM Ag assay (formerly AlereLAM), developed by Alere Inc. and now marketed by Abbott Laboratories, is a lateral flow immunochromatographic test designed for point-of-care use, particularly in resource-limited settings. It provides rapid, bedside detection of urinary LAM, with utility in diagnosing extrapulmonary TB cases that may yield negative sputum results by conventional methods such as GeneXpert (181). The performance of the Determine™ TB LAM test varies significantly based on patient characteristics, including the presence of TB symptoms, hospitalization status, and CD4+ T-cell count (185).

Since 2015, the WHO has recommended AlereLAM for TB screening in HIV-positive patients, particularly those with advanced immunosuppression (186). Meta-analyses report a pooled sensitivity of 42%, increasing to 54% among individuals with CD4+ counts below 100 cells/mm^3^, but falling to just 17% in those with higher counts (185). Despite its relatively modest sensitivity, AlereLAM remains a cost-effective diagnostic option in low-resource settings and contributes valuable clinical information, especially in advanced HIV infection (187, 188). The Fujifilm SILVAMP TB LAM assay (FujiLAM) represents a second-generation test that addresses several limitations of AlereLAM. FujiLAM incorporates high-affinity monoclonal antibodies and a silver amplification step to improve signal detection, thereby enhancing sensitivity and lowering the detection threshold for LAM in urine (189–192).

**Figure 4:**
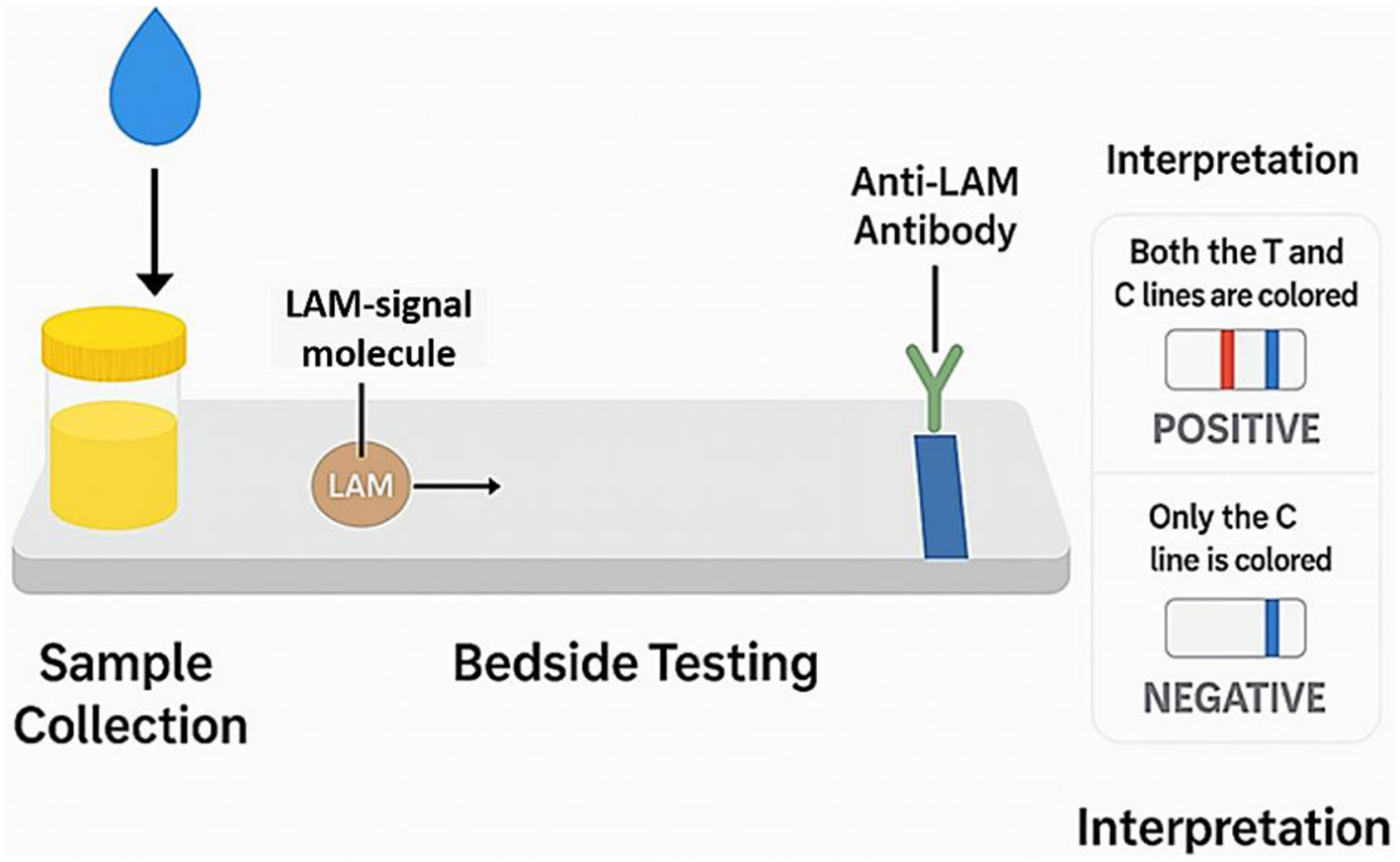
Schematic illustration of the LAM Assay principle. This diagram outlines the core steps of the LAM-based lateral flow immunoassay used for TB diagnosis. Following urine collection, LAM antigens bind to anti-LAM antibodies conjugated with a detection label. As the sample migrates along the test strip, the presence of LAM is indicated by a **colored test line**, while a control line ensures assay validity. The interpretation panel distinguishes between positive (both lines visible) and negative (only control line visible) results. This generic schematic avoids proprietary device branding and reflects the underlying principle applicable across LAM assay platforms.

Clinical studies have reported that FujiLAM can achieve diagnostic sensitivities of up to 85% in HIV-positive adults, with significantly higher specificity than AlereLAM (193–195). In a 2022 study conducted in Indonesia involving 62 patients, FujiLAM demonstrated a sensitivity of 61% and specificity of 92.31%, outperforming AlereLAM and other diagnostic methods in identifying extrapulmonary TB cases (196). In the same cohort, the general urine LAM test showed a sensitivity of 75% and specificity of 73.91%. Despite being three to four times more affordable than nucleic acid amplification tests (NAATs), AlereLAM's low sensitivity, particularly in patients with CD4+ T-cell counts above 200 cells/mm^3^, limits its broader clinical use (197). Additionally, ambiguities in the interpretation of AlereLAM results, as noted in the WHO 2019 guidelines, further complicate its clinical utility due to variability in host immune status and bacillary burden (185). A generalized schematic of the urine LAM assay principle is presented in Figure 4, illustrating the noninvasive detection of LAM antigens through a lateral flow immunoassay format. This process involves urine sample application, antigen-antibody binding, and visual interpretation of results via colored test and control lines.

### 3.5 Mass spectrometry technology

Matrix-assisted laser desorption ionization time-of-flight mass spectrometry (MALDI-TOF MS) has become an essential tool for microbial identification through protein profiling (198–200). While highly effective for many bacteria, its application to mycobacterial species has been more limited due to challenges in protein extraction from their lipid-rich, rigid cell walls (201, 202). These barriers complicate cell lysis and cytoplasmic protein release, both of which are critical for reliable MALDI-TOF MS analysis (203). Moreover, the slow growth rate and lower ribosomal content of MTBC, along with the need for biosafety level 3 (BSL-3) inactivation protocols, further hinder protein detection (204–206).

Recent advances in sample preparation have enhanced MALDI-TOF MS performance in mycobacterial diagnostics. Mechanical and chemical lysis techniques—particularly those employing silica/zirconia beads—have improved protein extraction efficiency (207), Bruker Daltonics' MycoEx protocol incorporates bead beating and acetonitrile extraction, showing improved performance in identifying mycobacteria, including *M. bovis* and *M. tuberculosis* strains (201, 205, 208, 209). Bacanelli et al. (210) demonstrated that high-powered homogenization (MycoLyser method), compared to vortexing (MycoEx), produced significantly higher Biotyper log scores (1.800 vs. lower scores), indicating enhanced identification accuracy.

Beyond species identification, MALDI-TOF MS is now applied for antimicrobial resistance profiling. The MassARRAY platform enables detection of hundreds of single nucleotide polymorphisms (SNPs) from 15 drug resistance-associated genes, generating comprehensive drug susceptibility profiles (211). In a clinical study of 201 pulmonary TB patients, Shi et al. (212) reported that MALDI-TOF MS had a detection rate of 94.3%, outperforming smear microscopy (43.2%), LAMP, 68.2% and Xpert MTB/RIF (85.2%). Notably, in culture-negative samples, MALDI-TOF MS still achieved a sensitivity of 78.8%, far exceeding other methods. Among rifampicin-resistant cases, MALDI-TOF MS identified 96.72%, compared to 81.97% with Xpert. These findings support MALDI-TOF MS as a robust and scalable platform for both rapid TB detection and drug resistance analysis. Continued refinement of sample processing protocols and broader clinical validation could solidify its role in frontline TB diagnostics, especially in settings requiring high-throughput, accurate, and rapid testing.

### 3.6 CRISPR-based diagnostics

The application of CRISPR (Clustered Regularly Interspaced Short Palindromic Repeats) and CRISPR-associated protein (Cas) systems in TB diagnostics represents a novel and promising molecular approach. CRISPR/Cas systems utilize sequence-specific cleavage activity, guided by short RNA molecules, to recognize and cleave complementary DNA sequences (213). In addition to their cis-cleavage capabilities at the target site, many Cas enzymes exhibit trans-cleavage activity—wherein abundant, non-target reporter oligonucleotides are repeatedly cleaved in proportion to target presence—allowing intrinsic signal amplification (214, 215).

This dual-cleavage mechanism enables ultra-sensitive detection of nucleic acids, including targets at low copy numbers or those differentiated by single nucleotide polymorphisms (SNPs) (216–219). Furthermore, CRISPR systems have demonstrated utility in detecting SNPs associated with drug resistance in *M. tuberculosis*, making them highly applicable for both diagnosis and antimicrobial resistance profiling (220). Their compatibility with portable, point-of-care platforms further enhances their diagnostic appeal (221–223).

Most CRISPR-based TB assays incorporate nucleic acid amplification (NAA), such as recombinase polymerase amplification (RPA) or LAMP, to enhance sensitivity (215, 224). These platforms can detect trace quantities of *M. tuberculosis* DNA with high accuracy in under an hour (225–227). However, total assay time often exceeds 60 min due to multi-step workflows (226, 228–232), which may also increase the risk of cross-contamination. Additionally, fluorescent signal detection in many CRISPR systems requires external devices, limiting field applicability in resource-limited settings until simpler readout systems become available. Lateral flow strips, however, offer promising visual alternatives for qualitative interpretation (227, 232, 233).

Most CRISPR-based TB diagnostics target multicopy genomic sequences such as IS6110 and IS1081, which are highly conserved and present in multiple copies across *M. tuberculosis* complex strains, thereby improving diagnostic sensitivity (225–227, 229, 232–234). Some systems have also been designed to detect mutations in the rifampicin resistance-determining region of the *rpoB* gene—mutated in over 95% of rifampicin-resistant and more than 78% of multidrug-resistant TB strains (228, 235, 236). Additional targets, such as 16S rRNA, rpsL, and gyrB, may also indicate TB presence or drug resistance. While genomic conservation across *M. tuberculosis* complex species exceeds 99% (237), IS6110 remains a universal and highly specific target for CRISPR-based detection platforms (238).

In 2023, Zhang et al. introduced a CRISPR/Cas12a-based system combined with recombinase-aided amplification for detecting *M. tuberculosis*, achieving rapid, equipment-free molecular identification (239). The entire workflow—from DNA extraction to signal detection—was completed within 2 h, including 20 min for extraction, 30 min for amplification, and 30 min for CRISPR-based readout. Compared to Xpert MTB/RIF (120 min) and conventional culture (up to 30 days) (240), the CRISPR approach offers significant time and cost advantages, with expenses estimated to be nearly 50% lower than standard PCR methods (241).

Despite its potential, several challenges remain. The variability in repeatability and the amplification duration may affect detection sensitivity and complicate quantification of bacterial load. Furthermore, the limitations of traditional culture-based reference standards (e.g., low positivity in older samples) suggest that aligning CRISPR results with clinical diagnoses may improve diagnostic accuracy (239). Nonetheless, CRISPR-based diagnostics represent a powerful emerging tool in the early and precise detection of TB and drug resistance mutations, particularly in resource-limited and high-burden settings.

### 3.7 The emerging role of artificial intelligence in TB diagnosis and resistance prediction

The integration of artificial intelligence (AI) and machine learning (ML) in TB diagnostics represents a transformative frontier in clinical microbiology and global health (242). These technologies are increasingly being used to optimize image interpretation, predict drug resistance, and integrate diverse diagnostic data streams for improved clinical decision-making. In microscopy, AI algorithms have demonstrated strong potential in automating the detection of AFB in stained sputum smears. For instance, convolutional neural network (CNN)-based models have been trained to identify AFB with high accuracy, thereby minimizing manual effort and inter-observer variability. Hwang et al. (243) developed a deep learning model that achieved over 96% sensitivity and 98% specificity for detecting TB-positive smears, enabling rapid, high-throughput analysis suitable for decentralized laboratories where trained personnel are limited.

In the domain of chest radiography, AI-driven computer-aided detection (CAD) systems such as CAD4TB, Lunit INSIGHT CXR, and qXR have become integral to TB screening programs in community and clinical settings. These systems analyze digital chest X-rays in real time and assign TB likelihood scores to assist in triaging suspected cases. Qin et al. (244) conducted a multicenter study demonstrating that CAD4TB version 6 performed comparably to expert radiologists in identifying TB, especially in asymptomatic and HIV-positive individuals. Similarly, the WHO evaluated multiple AI-based CAD tools and reported that Lunit and qXR achieved sensitivities exceeding 90% and met or surpassed the WHO's target product profile for TB triage tests (245). The latest versions of these systems have shown area under the curve (AUC) values nearing 0.90, supporting their deployment in mobile clinics and high-volume screening programs.

Beyond imaging, AI is playing a transformative role in the prediction of drug resistance using WGS data. ML models, including ensemble techniques like XGBoost, have been applied to genomic datasets to forecast resistance to first-line anti-TB drugs. Walker et al. (246) demonstrated that such models could predict resistance to rifampicin and isoniazid with over 90% accuracy, offering a rapid alternative to conventional phenotypic susceptibility testing. This innovation is particularly critical for timely management of multidrug-resistant TB. Furthermore, AI is being incorporated into clinical decision support systems that integrate diverse data inputs—including patient history, lab results, radiographic findings, and genetic profiles—to guide diagnosis and therapy in real time. These systems represent a move toward precision medicine in TB care.

**Figure 5:**
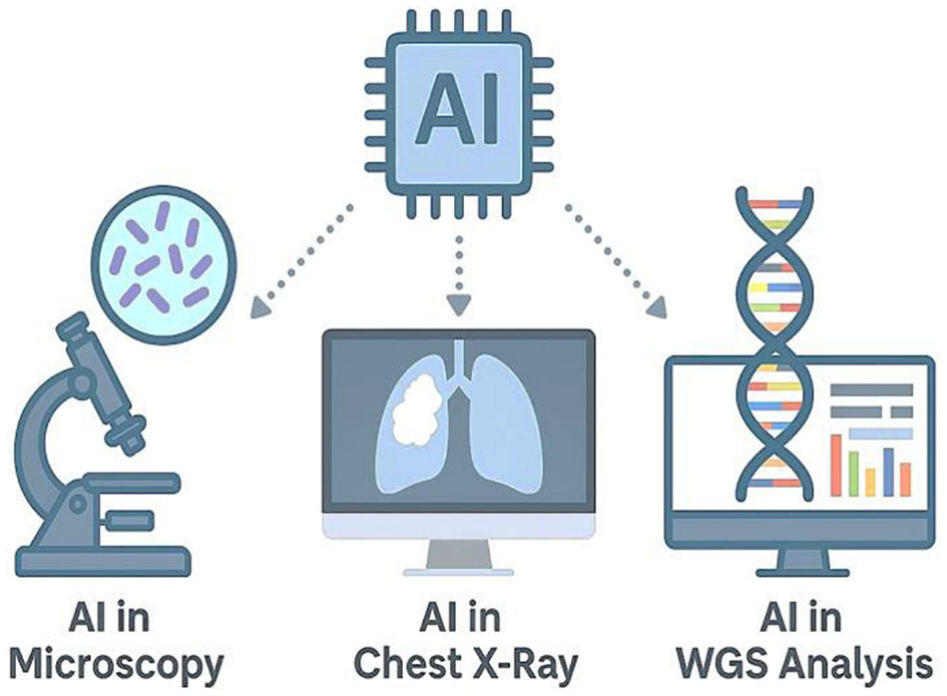
Artificial intelligence applications in tuberculosis diagnostics. This infographic illustrates the integration of AI across three major TB diagnostic platforms. **Left:** AI-assisted microscopy uses deep learning to detect AFB in smear samples. **Center:** AI-driven radiographic systems, such as CAD4TB, analyze chest X-rays for automated TB screening. **Right:** AI algorithms applied to WGS data enable rapid prediction of drug resistance mutations in *M. tuberculosis*. At the center, an AI microchip symbolizes the convergence of machine intelligence in advancing TB diagnostics across modalities.

Nonetheless, several barriers remain. These include the requirement for large annotated datasets, variability in model performance across populations, concerns over algorithm transparency, and data privacy regulations that complicate implementation. Additionally, infrastructure limitations and regulatory uncertainty pose challenges to the large-scale adoption of AI tools in TB programs. Despite these limitations, the expanding evidence base supports the transformative potential of AI in TB diagnostics. By improving detection speed, enhancing diagnostic precision, and enabling personalized treatment approaches, AI is poised to become a cornerstone of global TB control strategies. Figure 5 provides a visual summary of the integration of AI across key TB diagnostic platforms, including microscopy, chest radiography, and WGS.

### 3.8 Influence of host genetics and microbiome composition on TB susceptibility and treatment outcomes

Emerging research has highlighted the critical role of host genetic variation and microbiome composition in modulating susceptibility to TB, response to treatment, and risk of disease progression. These host-specific factors represent promising frontiers for the development of precision medicine approaches in TB management. Host genetics play a pivotal role in determining individual risk of TB infection, latency, and treatment response. Genome-wide association studies have identified multiple susceptibility loci, such as variants in the *TLR1, VDR, IFNGR1*, and *IL12B* genes, which affect immune recognition and cytokine signaling during *M. tuberculosis* infection. For instance, Curtis et al. (247) found that polymorphisms in HLA class II genes were significantly associated with progression from latent to active TB in diverse populations. Similarly, Quistrebert et al. (248) identified rare monogenic variants in TYK2 and STAT1 pathways linked to early-onset extrapulmonary TB, particularly in children.

Genetic differences also influence treatment outcomes. Polymorphisms in NAT2, which encodes N-acetyltransferase 2, affect isoniazid metabolism, leading to variations in drug efficacy and hepatotoxicity risk. In a meta-analysis by Huang et al. (249), slow acetylators had significantly higher risks of isoniazid-induced liver injury, suggesting the potential utility of pharmacogenomic-guided dosing. Beyond host DNA, the gut and lung microbiomes have emerged as important modulators of host immunity during TB infection and treatment. Dysbiosis—characterized by reduced microbial diversity and loss of commensal species—is commonly observed in TB patients, potentially impairing immune homeostasis. Naidoo et al. (250) demonstrated that anti-TB therapy induces long-term gut microbiome alterations, including depletion of *Clostridiales* and *Bifidobacterium* spp., which correlated with systemic inflammation and altered immune profiles.

**Figure 6:**
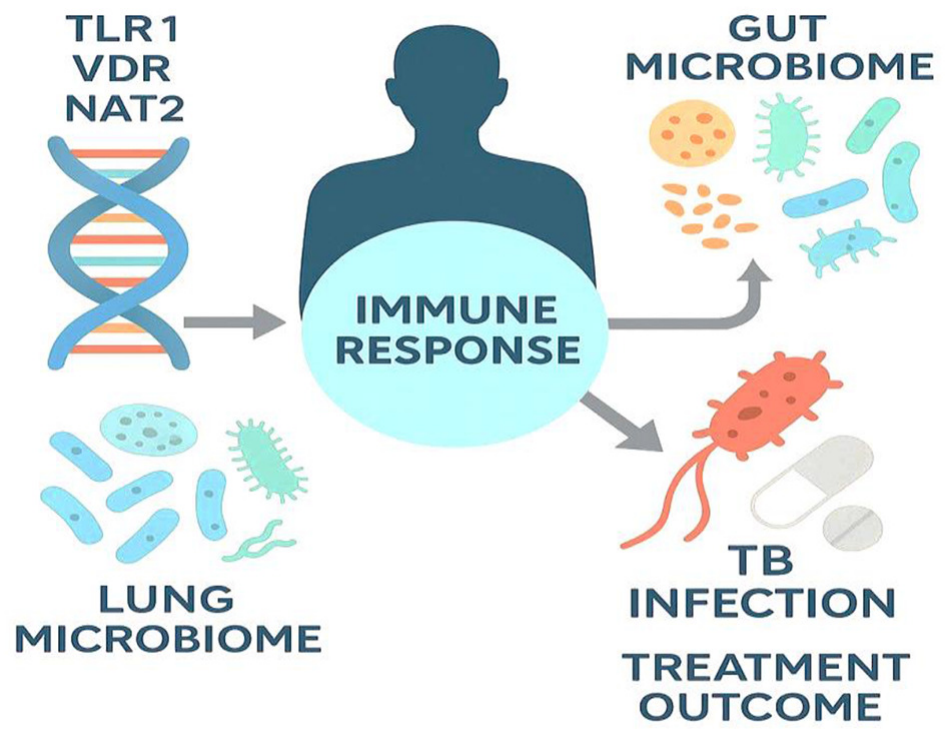
Host genetics and microbiome interactions shaping TB outcomes. The schematic highlights how host genetic variants (e.g., *TLR1, VDR, NAT2*) and the composition of the gut and lung microbiomes modulate the immune response to *M. tuberculosis*. These host-specific factors influence infection risk, disease severity, and the success of pharmacologic therapy, underscoring the potential of precision-guided TB management that integrates genomics and microbiome profiling.

Alterations in the lung microbiota have been increasingly linked to TB severity. Studies show that patients with active TB exhibit reduced microbial diversity and a shift from commensal genera like *Streptococcus* and *Prevotella* to potentially pathogenic taxa such as *Rothia* and *Veillonella*, reflecting a dysbiotic state that may worsen inflammation and lung damage (251). These findings suggest that the lung microbiome not only influences host immunity but may also serve as a biomarker or therapeutic target in TB management. Figure 6 illustrates how host genetic factors and microbiome composition interact to influence TB susceptibility, progression, and treatment outcomes.

## 4 Treatment options of TB

Accurate diagnosis of TB is crucial for guiding treatment strategies, determining drug selection, and defining therapy duration. Techniques such as sputum smear microscopy, molecular assays, and immunological tests are essential for distinguishing between drug-sensitive and drug-resistant strains of *M. tuberculosis* (252). Effective diagnostics improve therapeutic outcomes, reduce transmission, and enhance public health interventions. While 85% of patients with drug-sensitive TB benefit from current treatment regimens, only 57% of those with drug-resistant TB achieve successful outcomes (253), highlighting the need for optimized, cost-effective oral therapies (254).

### 4.1 Therapy for drug-susceptible TB

The standard 6-month regimen for drug-susceptible tuberculosis, established through decades of clinical trials by the British Medical Research Council, remains the global benchmark (255). It comprises a 2-month intensive phase using rifampicin, isoniazid, and pyrazinamide, followed by a 4-month continuation phase with rifampicin and isoniazid. The inclusion of pyrazinamide significantly reduced the treatment duration from 9 to 6 months, enhancing global treatment adherence (255). While this short-course chemotherapy has saved millions of lives, its 6-month duration presents logistical and adherence challenges for both patients and TB programs (256). To address this, efforts have focused on shortening therapy without compromising efficacy. Fluoroquinolones such as moxifloxacin and gatifloxacin have demonstrated accelerated sputum sterilization (257, 258), but three pivotal phase III trials in 2014 failed to prove non-inferiority compared to the standard regimen (259, 260). Nonetheless, subgroup analyses suggest that certain patient populations may benefit from abbreviated regimens (261, 262).

Rifamycin optimization is another approach to treatment shortening. High-dose rifampicin (up to 40 mg/kg/day) is well tolerated and may improve sterilizing activity (263), particularly for TB meningitis. A recent phase III trial demonstrated that a 4-month regimen containing rifapentine, isoniazid, pyrazinamide, and moxifloxacin was non-inferior to the standard 6-month protocol, prompting provisional WHO endorsement in 2022 for drug-susceptible pulmonary TB (264, 265). Newer agents such as pretomanid, a nitroimidazole compound, have also shown promise. A phase II trial reported faster sputum culture conversion compared to standard therapy, though its use was associated with a higher incidence of hepatotoxicity, especially when combined with pyrazinamide (266).

In pediatric cases, shorter therapy may also be effective (267). The SHINE trial, a phase III open-label study, revealed that 16-week regimens were non-inferior to 6-month protocols in children with non-severe, smear-negative TB (268). Similarly, the 2023 TRUNCATE-TB trial evaluated 2-month regimens in adults. Among 674 participants, the 8-week bedaquiline-linezolid group achieved non-inferiority with no significant safety concerns, while the rifampin-linezolid group did not meet the non-inferiority margin (269). These findings highlight a growing opportunity for individualized, shorter-course therapy in selected cases of drug-susceptible TB.

### 4.2 Isoniazid-resistant, rifampicin-susceptible TB treatment

Isoniazid has served as a cornerstone for both active and latent TB treatment for over five decades (270). However, increasing rates of isoniazid resistance have become a major clinical concern. According to the WHO, ~7.4% of newly diagnosed TB patients and 11.4% of previously treated individuals are resistant to isoniazid while remaining susceptible to rifampicin—a condition classified as isoniazid-resistant, rifampicin-susceptible TB (Hr-TB) (271). This form of resistance is more common than multidrug-resistant TB (MDR-TB), highlighting the urgency of appropriate therapeutic strategies. Failure to manage isoniazid resistance according to guidelines significantly elevates the risk of acquiring additional resistance, particularly to rifampicin and other first-line agents (272, 273). Recognizing this, the WHO currently recommends a 6-month regimen comprising rifampicin, ethambutol, pyrazinamide, and levofloxacin for the treatment of Hr-TB. However, this recommendation remains conditional due to the limited strength of supporting evidence and the absence of randomized controlled trials validating its efficacy (272, 274, 275).

Alternative international guidelines, such as those from European and American expert panels, suggest using pyrazinamide only during the initial 2 months of therapy in selected cases to minimize hepatotoxicity (276). In scenarios where fluoroquinolone resistance is confirmed or suspected, a 6-month combination of rifampicin, ethambutol, and pyrazinamide is typically administered. However, such recommendations are largely based on expert consensus rather than high-quality clinical data. Additionally, the WHO has not yet addressed the potential role of high-dose isoniazid in managing Hr-TB, despite emerging evidence suggesting that its effectiveness may depend on specific resistance-conferring mutations and individual acetylator status (277). Most existing data on Hr-TB treatment stem from observational studies rather than randomized trials, underscoring the critical need for rigorously designed clinical research to inform optimal therapeutic regimens (278).

In a comprehensive analysis involving WHO surveillance data from 156 countries between 2003 and 2017, Dean et al. (271) estimated that Hr-TB prevalence was 7.4% in newly diagnosed cases and 11.4% in previously treated patients. Resistance to pyrazinamide and levofloxacin was comparatively rare, being reported in only 1.8% and 5.3% of the assessed countries, respectively. Whole-genome sequencing (WGS) data from 4,563 clinical samples showed that 78.6% of isoniazid-resistant strains harbored mutations in the *katG* gene, particularly the Ser315Thr substitution, which is known to confer high-level resistance. A recent 2023 study by Liu et al. (279) explored the genetic mechanisms of isoniazid resistance in *M. tuberculosis* isolates from China. Out of 4,922 clinical isolates analyzed using WGS, 384 (7.8%) exhibited resistance to isoniazid while remaining rifampicin-sensitive. The *katG* Ser315Thr mutation was observed in 63.0% of cases, and *fabG1* mutations were found in 29.9%. Importantly, resistance rates for pyrazinamide (0.8%), ethambutol, fluoroquinolones (2.3%), and amikacin (0.5%) were low, whereas resistance to streptomycin was significantly higher at 39.6%. These findings support the use of rifampicin, ethambutol, pyrazinamide, and levofloxacin as an effective combination regimen in managing Hr-TB, provided resistance to companion drugs is excluded.

### 4.3 Multidrug-resistant and rifampicin-resistant TB treatment

Multidrug-resistant tuberculosis (MDR-TB) and rifampicin-resistant TB (RR-TB) continue to pose significant public health challenges globally, with an estimated 450,000 new cases of RR-TB expected in 2021 (280). The global treatment success rate for patients treated for MDR/RR-TB improved from ~50 % in 2012 to 60 % in 2019, rising further to 63 % in 2020 (281). However, it is concerning that 15% of patients diagnosed with MDR/RR-TB do not survive. In December 2022, the WHO released the WHO Consolidated Guidelines on TB, Module 4: Treatment—Drug-Resistant TB Treatment, which replaces the 2020 edition and expands on previous recommendations (265). The 2022 update outlines seven critical areas relevant to the treatment of MDR-TB (282). These areas include strategies for managing MDR/RR-TB, handling isoniazid-resistant TB, monitoring patient responses to therapy, determining the optimal timing for initiating antiretroviral therapy in patients co-infected with HIV, and considering surgical interventions for patients with MDR/RR-TB (265).

The 2022 guidelines recommend two new treatments for MDR/RR-TB (283). First, a 6-month regimen of bedaquiline, pretomanid, linezolid (600 mg), and moxifloxacin is proposed as an alternative to longer regimens (284). Second, an all-oral regimen for 9 months is advised if fluoroquinolone resistance is eliminated, though extended regimens may still be an option (285). In 2018, more than 12,000 patients with MDR/RR-TB (286, 287) experienced a reduction in treatment duration from 18–20 months to 9–12 months. The STREAM Stage 2 trial revealed that 71% of patients on injectable regimens and 83% in the all-oral group had positive outcomes (288), with lower rates of grade 3/4 hearing loss in the all-oral group (2% vs. 9%).

The WHO recommended a 9–12 month bedaquiline regimen for TB cases without fluoroquinolone resistance (265). The TB-PRACTECAL study (289) demonstrated that the BPaLM regimen (bedaquiline, linezolid, pretomanid, moxifloxacin) achieved 89% positive outcomes with fewer side effects than standard treatment, leading to the study's early termination. The NExT trial further reduced treatment duration to 6 months with bedaquiline, linezolid, and fluoroquinolones (290). An interim analysis of the BEAT Tuberculosis trial reported 87% effectiveness with a 6-month regimen (291). The MDR-END trial accomplished 75% success with a non-bedaquiline regimen, showing non-inferiority to the previous 20–24-month treatment duration recommended by the WHO in 2014 (292). Progress in managing MDR/RR-TB is hindered by inadequate drug resistance testing (293). The lack of standardized testing limits diagnostics and undermines clinician trust, while high drug costs restrict availability in many countries (294).

### 4.4 Treatment of multidrug-resistant/rifampicin-resistant and fluoroquinolone-resistant TB

The treatment of pre-extensively drug-resistant tuberculosis (pre-XDR-TB)—which includes multidrug-resistant (MDR) and rifampicin-resistant (RR) TB with additional fluoroquinolone (FQ) resistance—remains a major clinical challenge. This is largely due to limited therapeutic options, high drug costs, frequent adverse effects, and historically poor outcomes (256). The BEAT-India trial demonstrated promising results, achieving a 91% treatment success rate among 153 patients treated with a 6–9-month regimen containing bedaquiline, linezolid (600 mg daily), clofazimine, and delamanid. Despite the high efficacy, linezolid-related toxicities were significant, though some patients tolerated a reduced dose of 300 mg (295). The NiX-TB trial evaluated a three-drug BPaL regimen (bedaquiline, pretomanid, and linezolid 1,200 mg daily) for 6 months, reporting a 90% success rate but with high rates of adverse effects-−81% developed peripheral neuropathy, and 48% experienced myelosuppression. The subsequent ZeNix trial tested lower linezolid doses (600 or 1200 mg for 2 or 6 months) and showed success rates between 84% and 93%, with improved safety at the 600 mg dose (296, 297).

Based on these findings, the WHO recommends the BPaL regimen for the treatment of fluoroquinolone-resistant TB (298). However, evidence supporting the 600 mg dose recommendation is still evolving, and the optimal duration and dosage of linezolid remain subjects of ongoing investigation (299, 300). A retrospective cohort study conducted by Lee et al. in South Korea between 2005 and 2017 included 129 MDR-TB patients, of whom 30.2% were FQ-resistant and 69.8% were FQ-sensitive. Linezolid was the most frequently prescribed drug in the FQ-resistant group (51.3%), followed by bedaquiline (20.5%) and delamanid (10.3%). Although no significant difference in treatment success was observed between FQ-sensitive and FQ-resistant patients, the study emphasized that individualized regimens incorporating new drugs can improve treatment outcomes for difficult-to-treat TB cases (301).

In support of this approach, Nehru et al. conducted a genomic epidemiological study using the WHO-endorsed GenoType MTBDRsl Ver 2.0 assay to evaluate fluoroquinolone resistance in various TB subtypes. The study found FQ resistance in 33% of MDR-TB cases, 16.5% of RR-TB cases, and 5.4% of non-MDR-TB isolates. The most prevalent mutation was D94G in the *gyrA* gene, accounting for 49.5% of resistance-related mutations. Alarmingly, 5.12% of isoniazid mono-resistant isolates also exhibited FQ resistance, and isolates harboring the S450L mutation in the *rpoB* gene were associated with increased risk. These findings underscore the critical need for routine resistance testing before initiating treatment, especially in high-burden regions (302).

### 4.5 Host-directed therapies

**Figure 7:**
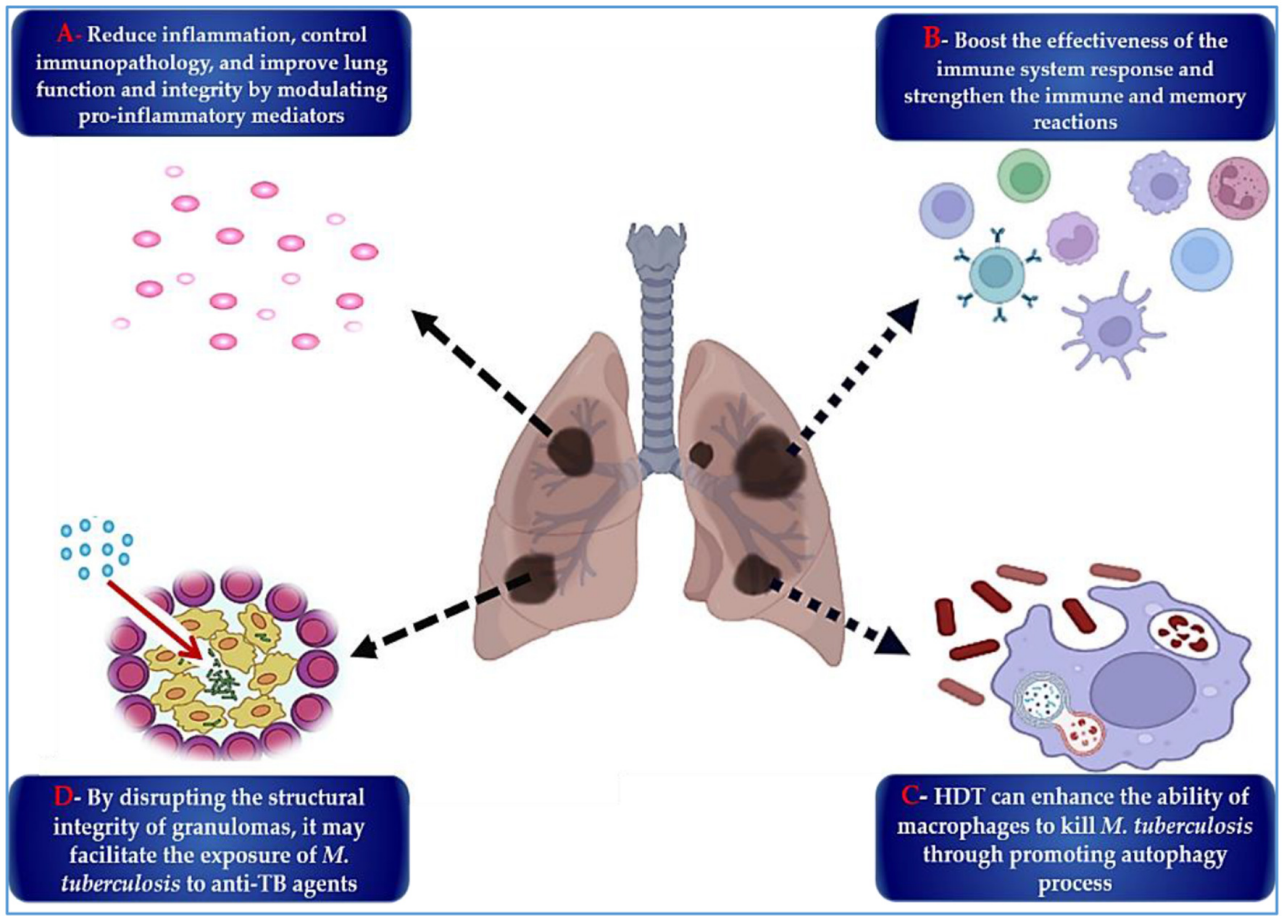
The key HDTs that may enhance the efficacy of *M. tuberculosis* treatment are as follows: **(A)** Regulating inflammatory mechanisms and mediators is crucial for reducing inflammation and preventing lung tissue damage, ultimately enhancing lung integrity. **(B)** Strengthening the host immune and memory responses is essential for overall health. **(C)** Enhancing host bactericidal mechanisms, such as macrophage-mediated killing of *M. tuberculosis* and inhibiting bacillary growth, is vital for an effective immune response. **(D)** The disintegration of granulomas and the release of *M. tuberculosis* bacilli increase exposure to anti-TB medications.

In the ongoing pursuit of more effective TB treatments, two parallel strategies have gained prominence: the development of antimycobacterial drugs and host-directed therapies (HDTs), which aim to enhance the host's immune response (303, 304). HDTs reduce disease burden and potentially counteract antibiotic resistance by minimizing reliance on antimicrobials and enhancing the efficacy of existing drugs (305). These therapies often involve the repurposing of immune-modulating agents that improve pathogen clearance, suppress harmful inflammation, and limit tissue destruction (304, 305). HDT targets key aspects of TB immunopathogenesis, including excessive inflammation (306), host metabolic processes (307), and the immune evasion mechanisms employed by *M. tuberculosis* (308). By modulating these host factors, HDTs offer an adjunctive approach to conventional anti-TB treatment. As illustrated in Figure 7, HDTs exert their effects via four primary pathways: reducing lung inflammation and tissue damage; enhancing antimicrobial immune responses; promoting direct bactericidal activity; and disrupting granulomas to expose intracellular bacteria to host defenses and antibiotics.

The overarching objective of HDT is to amplify protective host responses while limiting detrimental ones that contribute to bacterial persistence and lung pathology (309). If successfully implemented, HDT may improve treatment adherence, reduce treatment duration, lower the risk of resistance development, and ultimately improve cure rates with fewer long-term complications (310). Many HDTs currently under investigation are repurposed drugs being tested in preclinical or clinical trials as adjuncts to standard therapy. Notably, agents such as acetylsalicylic acid and statins offer potential advantages due to their established safety profiles and affordability, which may facilitate rapid clinical integration if proven effective (311). Moreover, HDTs may reduce the dependency on prolonged use of repurposed antibiotics such as oxazolidinones and fluoroquinolones, thereby lowering the risk of resistance development in *M. tuberculosis* and other pathogens (312). Additionally, their anti-inflammatory properties may mitigate pulmonary damage and improve patient outcomes (309). Nonetheless, HDT development faces key challenges. *In vitro* studies remain essential for initial pharmacological screening (313), while animal models provide valuable insights into immune modulation, treatment efficacy, and disease progression. These models help assess therapeutic effects on bacterial load, tissue pathology, and survival (314). Ultimately, findings from experimental models must be validated in clinical trials to determine real-world applicability and address safety, efficacy, and regulatory considerations (315).

### 4.6 Digital health tools and telemedicine in TB management

The emergence of digital health technologies and telemedicine platforms has opened new avenues for improving TB care, particularly in settings with limited healthcare access or disrupted services. These tools are increasingly utilized for treatment adherence monitoring, contact tracing, remote diagnostics, and patient education—forming a critical component of modern TB control strategies. One of the most impactful digital innovations is the digital adherence technology (DAT) platform, which includes tools such as 99DOTS, evriMED smart pillboxes, and video directly observed therapy (vDOT) (316). These systems track medication intake in real-time and alert healthcare providers to missed doses. A multicountry evaluation by Thomas et al. (317) found that 99DOTS, a low-cost cellphone-based system, improved medication adherence among TB patients in India, with 93% of doses recorded on the platform and significantly fewer missed doses compared to standard DOT.

Similarly, vDOT platforms like SureAdhere have been adopted by TB programs in the U.S. and Europe, enabling patients to record and submit videos of themselves taking their medications. A randomized controlled trial by Story et al. (318) demonstrated that vDOT was non-inferior to in-person DOT, with higher patient satisfaction and reduced logistical burden on health workers. In rural or crisis-affected regions, telemedicine has played a vital role in bridging access gaps. Real-time video consultations, chat-based triage systems, and remote expert panels facilitate timely diagnosis and clinical decision-making. For example, during the COVID-19 pandemic, the TB REACH initiative implemented a telehealth-supported TB triage model in Pakistan that reduced diagnostic delays by over 40% (319).

Mobile applications (e.g., Nikshay in India, TB eHealth in Uganda) now offer centralized digital platforms to track patient records, laboratory results, and treatment progress, allowing health authorities to monitor outbreaks in real time and improve linkage to care (320). These platforms also integrate AI-based analytics to identify high-risk patients and predict treatment interruptions. Despite their potential, digital TB tools face challenges such as poor internet connectivity, digital illiteracy, privacy concerns, and lack of integration into national health information systems. Nonetheless, as part of the WHO's End TB Strategy, digital health is recognized as a cornerstone for improving TB program efficiency, enhancing patient-centered care, and achieving universal health coverage.

### 4.7 Advances in TB vaccine development: challenges and new frontiers

The development of an effective TB vaccine remains a pressing global health priority. While the BCG vaccine, first introduced in 1921, is still in use, its protective efficacy is highly variable—especially against pulmonary TB in adolescents and adults (321). With increasing rates of drug-resistant TB and the global burden of latent infections, the need for improved preventive strategies has gained renewed momentum. Recent advances in immunology, systems biology, and vaccine technology have led to the emergence of over 17 vaccine candidates currently in various phases of clinical development (322). Among the most promising candidates is M72/AS01E, a subunit vaccine developed by GSK and Aeras. In a pivotal Phase IIb trial involving 3,573 latently infected adults in Kenya, South Africa, and Zambia, the vaccine demonstrated ~50% efficacy in preventing active TB over a 3-year follow-up period (323). This result marked the first successful demonstration of vaccine-mediated protection against TB in latently infected adults and has led to plans for a large-scale Phase III efficacy trial.

VPM1002, a recombinant BCG vaccine engineered to express listeriolysin O and delete the ureC gene, has shown enhanced immunogenicity and safety in comparison to traditional BCG. Phase II trials conducted in India and South Africa have reported favorable results, and Phase III trials are currently underway in both infants and adults (324). Another advanced candidate is MTBVAC, the first live-attenuated vaccine derived directly from *M. tuberculosis* rather than *M. bovis*. Developed by Biofabri and the University of Zaragoza, MTBVAC has completed Phase I and II studies with promising safety and immunogenicity profiles. It is currently in Phase III trials in multiple countries, including high-burden settings in Africa (325). In addition to these, ID93+GLA-SE, a protein–adjuvant vaccine, has shown robust immune responses in early trials and completed Phase IIa evaluation (326).

Ad5Ag85A, a recombinant adenoviral-vectored vaccine, is being evaluated for its ability to enhance mucosal immunity—a key defense mechanism in pulmonary TB. Notably, mRNA-based TB vaccines, such as those developed by BioNTech, have recently entered Phase I clinical testing following strong preclinical evidence of immunogenicity and protection (327). These candidates represent a promising shift toward faster, more adaptable vaccine development strategies. A summary of the leading TB vaccine candidates currently in clinical development is provided in Table 1. These candidates span diverse technological platforms and clinical stages, underscoring the momentum and innovation in the TB vaccine pipeline.

**Table 1:** Leading Tuberculosis Vaccine Candidates in Clinical Development.

**Vaccine candidate**	**Platform**	**Clinical phase**	**Key findings**	**Key references**
M72/AS01E	Subunit (Mtb32A–Mtb39A + AS01E adjuvant)	Completed Phase IIb; Phase III planned	~50% efficacy in latent TB adults	(323)
VPM1002	Recombinant BCG (listeriolysin O; ΔureC)	Ongoing Phase III	Improved safety/immunogenicity	(324)
MTBVAC	Live-attenuated M. tuberculosis strain	Ongoing Phase III	Safe, immunogenic in infants and adults	(325)
ID93+GLA-SE	Protein–adjuvant (ID93 + GLA-SE)	Completed Phase IIa	Robust T-cell responses	(333)
Ad5Ag85A	Recombinant adenoviral vector expressing Ag85A	Phase I completed	Safe and immunogenic in BCG-primed adults; induced polyfunctional T cells	(334)
mRNA-based vaccines	Synthetic mRNA-based platforms	Phase I (initiated 2023)	Preclinical protection; early trials begun	(327)

Despite the growing diversity of vaccine strategies, several challenges continue to hinder progress. These include the absence of validated immune correlates of protection, the high costs and lengthy timelines associated with efficacy trials, and the biological complexity of latent TB infection. Furthermore, regulatory uncertainties, limited commercial incentives, and diminished prioritization in high-income countries where TB incidence is low contribute to underinvestment in vaccine development (328). Nonetheless, this surge of innovation marks a turning point in TB vaccine research. Continued support for translational science, robust public–private partnerships, and equitable implementation planning will be essential. With strategic investments, the global community may finally be on the cusp of introducing the first new TB vaccine in over a century—one that can significantly alter the trajectory of the epidemic by preventing disease, reducing relapse, and mitigating the spread of drug-resistant strains.

## 5 Future directions for diagnosis and treatment of TB

Significant strides have been made in the diagnosis and treatment of TB, driven by advances in point-of-care technologies, innovative therapeutics, and integrated public health frameworks. The incorporation of molecular assays, immunodiagnostics, mass spectrometry, and CRISPR-based platforms has markedly improved diagnostic precision and speed, enabling real-time detection of *M. tuberculosis* and its resistance profiles. These advancements support earlier and more targeted treatment initiation, particularly in high-burden settings. Therapeutically, the emergence of host-directed therapies aimed at modulating the immune response holds promise for enhancing treatment efficacy, reducing tissue damage, and shortening therapy duration. In parallel, vaccine development has progressed with the design of novel candidates intended to bolster both preventive and therapeutic immunity against TB.

Public health strategies are also evolving to emphasize community-centered approaches and integrated care models, particularly for individuals with co-morbid conditions such as HIV and diabetes. These models aim to improve patient retention, treatment adherence, and overall outcomes. Moreover, increased global investment in TB research and the formation of public-private partnerships are accelerating innovation, facilitating the translation of scientific breakthroughs into scalable interventions. Together, these developments signify a shift toward a more holistic and forward-thinking TB control paradigm—one that prioritizes early detection, personalized treatment, and equitable access to care in the global effort to reduce TB incidence and mortality.

## 6 Implementation challenges and cost-effectiveness of advanced TB technologies

**Figure 8:**
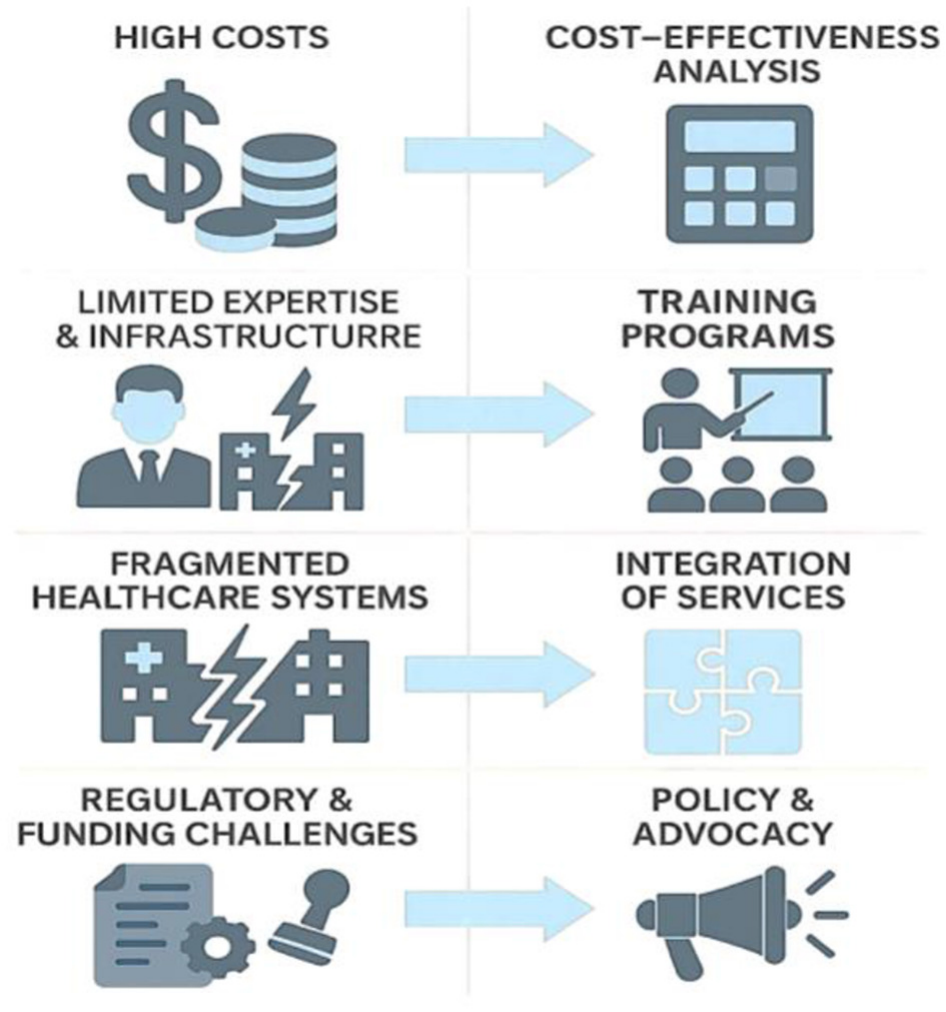
Implementation challenges and strategic solutions for advanced TB technologies. This infographic outlines four major barriers to implementing innovative TB diagnostics and treatments—high costs, limited infrastructure and workforce, fragmented healthcare systems, and regulatory or funding hurdles—and pairs each with corresponding solutions. These include cost-effectiveness analysis, training programs, service integration, and policy advocacy, all of which are critical to ensuring the real-world success of TB innovations in diverse health system contexts.

The effective integration of diagnostic and therapeutic innovations into TB control programs hinges not only on technological readiness but also on their cost-effectiveness, health system compatibility, and equitable access—key concerns addressed by implementation science (Figure 8) (329). This discipline evaluates how well evidence-based tools function in real-world clinical environments, particularly in LMICs. Despite the promise of technologies such as WGS, CRISPR-based diagnostics, and new regimens like BPaLM, their widespread adoption remains limited. Logistical, infrastructural, and financial constraints often impede implementation. For example, although WGS provides comprehensive and rapid drug-resistance profiling, its use in resource-limited settings is curtailed by high setup costs, the requirement for skilled personnel, and limited bioinformatics infrastructure. A study in the Kyrgyz Republic reported an initial capital investment of over $220,000, with early per-sample costs averaging $277—figures that present major barriers to scale-up (149).

Cost-effectiveness analyses are vital for policy-making in settings with limited resources. Vassall et al. (330) found that molecular diagnostics like Xpert MTB/RIF outperform smear microscopy in both effectiveness and cost, particularly in high-burden settings by reducing diagnostic delays. Similarly, Isaacs et al. (331) showed that digital adherence tools—such as video-observed therapy and SMS reminders—can be cost-saving, especially when accounting for patient-incurred costs, although effectiveness varies by context. Healthcare system fragmentation, especially in countries with parallel public and private sectors, further complicates implementation. Pai and Pakdil (332) noted that expanding diagnostic access without concurrent investments in healthcare quality, supply chains, workforce capacity, and accountability mechanisms yields limited benefits.

Sustainable adoption is also hindered by regulatory barriers, fragmented funding streams, and donor-driven priorities that may neglect long-term system strengthening. As a result, validated technologies like Line Probe Assays or video-DOT platforms often remain underutilized. To overcome these challenges, national TB strategies must incorporate implementation research, health technology assessments, and economic modeling. Embedding these components in TB innovation pipelines will help ensure that emerging tools are not only scientifically effective but also affordable, scalable, and contextually appropriate for diverse health systems.

## 7 Conclusions

Despite over a century of medical progress, tuberculosis remains a formidable global health challenge, exacerbated by the rise of antimicrobial resistance and the limitations of existing diagnostic and therapeutic tools. This review has highlighted how recent innovations—ranging from advanced molecular diagnostics and next-generation sequencing to novel therapeutics and promising vaccine candidates—are reshaping the landscape of TB control. However, technological advancement alone is insufficient. The true impact of these innovations depends on their integration into health systems, especially in LMICs where the burden is highest. Effective implementation must be guided by cost-effectiveness analyses, health equity considerations, and robust infrastructure development. Furthermore, the urgency to overcome regulatory, logistical, and funding barriers cannot be overstated if we are to bridge the gap between scientific discovery and public health impact. Encouragingly, the TB vaccine pipeline has expanded significantly, offering hope for a long-overdue replacement or complement to the BCG vaccine. To turn the tide against TB, a multifaceted approach is essential—one that harmonizes scientific innovation, political will, cross-sectoral partnerships, and community engagement. Only through such coordinated global efforts can we hope to achieve the goals of the End TB Strategy and finally relegate this ancient disease to history.
